# An Ethnobotanical study of medicinal plants in Taşköprü (Kastamonu–Turkey)

**DOI:** 10.3389/fphar.2022.984065

**Published:** 2022-10-20

**Authors:** Ismail Senkardes, Ahmet Dogan, Gizem Emre

**Affiliations:** Department of Pharmaceutical Botany, Faculty of Pharmacy, Marmara University, Basibuyuk-Istanbul, Turkey

**Keywords:** ethnobotany, medicinal plants, Taşköprü, Kastamonu, Turkey

## Abstract

This pharmaceutical ethnobotanical research was carried out in Taşköprü District in Kastamonu, in northern Anatolia. It assembles the elaborations of plants used as folk medicines, and the ethnopharmacological data collected in the course of in-person semi-structured interviews with an open-ended questionnaire. The study’s aims were two-fold: gathering and identifying plants that the local inhabitants use therapeutically, and recording information related to traditional folk medicine (primarily for humans, and if extant for animals). The plants were gathered during several outings between May 2016 and July 2018. The organization of the data was based on the use-reports (UR) and was done according to the ICPC-2 classification. In addition, cultural importance index (CI) and informant consensus factor (F_IC_) calculations were made for the data collected. The research identified 101 plant taxa of 31 families used in folk medicine. Of these, 89 were wild and 12 were cultivated taxa. In total, 499 medicinal uses were determined. The CI values indicated that the most significant medicinal plant specimens were *Pinus nigra* subsp. *pallasiana* (0.78), *P. sylvestris* var. *hamata* (0.75) and *Plantago lanceolata*, *P. major* subsp. *intermedia* and *P. major* subsp. *major* (0.58 each). The most prevalent families were Asteraceae (2.14), Rosaceae (1.93), Pinaceae (1.81) and Plantaginaceae (1.74). Respiratory system (0.95), skin and subcutaneous tissue (0.94), nervous system (0.92) and circulatory system disorders (0.88) and ethnoveterinary uses (0.89) had the highest F_IC_ values. The most frequently used preparation process was observed to be decoction (38.4%) and the most commonly utilized plant parts were aerial (21%). Along with recording 20 plant taxa as medicinal plants for the first time, this study documented a total of 303 new therapeutic uses. This study concludes with the finding that traditional knowledge of medicinal plants remains prevalent in Ta rticularly among its rural inhabitants.

## Introduction

Popular knowledge of the plants we use comes from millennia of experience. The knowledge that ancient civilizations possessed of plant use was widespread. Plants were our main therapeutic agents until the mid-19th century, and their medicinal functions remain relevant today (Camejo-Rodrigues et al., 2003). Based on their cultural experiences of their particular ecosystems, indigenous peoples across the globe have developed and maintained distinctive world-views that regulate the myriad relations among humans, non-humans, and other-than-humans. Such world-views and relations have come to comprise a system generally recognized as indigenous knowledge or as traditional knowledge (Bruchac, 2014). However, the increasing pace of the loss of global biodiversity is cause for alarm (Aswani et al., 2018). Equally alarming is the pace of the loss of traditional knowledge throughout the world as the long-established homelands of indigenous peoples are assailed by unfettered development (Ramirez, 2007). Thus, the primary focus of ethnobotany is now to protect humanity’s traditional knowledge of the natural world’s flora (Mattalia et al., 2020). The usage of plants in folk medicine has been documented world-wide by ethnobotanists (Karakose et al., 2019). Such ethnobotanical studies serve to preserve traditional knowledge and to facilitate the development of new drugs (Kathambi et al., 2020). These drugs, with their low cost, ease of availability and relatively few side effects, can complement or offer alternatives to existing therapies for a variety of diseases (Feng et al., 2019). Rightly so, medicinal plants are now recognized as local treasures of global importance, and as playing a key role in the lives of people who live in rural areas–especially in remote areas with restricted access to modern healthcare (Terzioglu and Coskuncelebi, 2021).

In Turkey and around the world, there is great untapped potential in investigative surveys of medicinal plants. These surveys provide disciplines such as complementary medicine, phytotherapy, pharmacology and veterinary medicine with valuable information. Demand for these ethnobotanical studies has been increased by the impulse in both developed and developing countries to use traditional methods to treat diseases (Akbulut et al., 2022). It can be said that nearly 80,000 of the approximately 374,000 flowering plants in the world are used for medicinal purposes (Schippmann et al., 2006; Christenhusz and Byng, 2016). In addition, Turkey has hosted many civilizations, and they have left rich cultural, social, and ecological heritages (Kendir and Güvenç, 2010).

Owing to its physical structure, different ecological zones, geographical variations, and diverse climates, Turkey’s flora is very rich. It comprises about 12,975 plant taxa, of which 4,157 are endemic (Ozhatay et al., 2019), and the endemism rate has been updated to 32% (Karakose, 2022b). With this diversity of flora, Turkey has rich herbal medicine resources (Ozhatay et al., 2012).

As well as documenting human interactions with medicinal plants, ethnobotanical studies identify plants which play an important role in the health of animals. The last 35 years have seen research, documentation and evaluation of traditional ethnoveterinary practices across the globe (McCorkle, 1986; Yigezu et al., 2014). In Turkey, a recent surge of studies with a focus on documenting traditional ethnoveterinary knowledge has yielded valuable information about ethnoveterinary practices (Erarslan and Kultur, 2019; Güler et al., 2021; Kazanci et al., 2021; Akbulut, 2022).

Many researchers (Sezik et al., 1991; Sezik et al., 1992; Fujita et al., 1995; Yazıcıoglu and Tuzlaci, 1996; Sezik et al., 1997; Yesilada et al., 1999; Tuzlaci and Tolon, 2000; Uzun et al., 2004; Ecevit Genc and Ozhatay 2006; Ezer and Mumcu Arisan, 2006; Turkan et al., 2006; Cansaran et al., 2007; Kultur, 2007; Tuzlaci and Alparslan, 2007; Akgul, 2008; Koyuncu et al., 2009; Koca and Yildirimli, 2010; Tuzlaci et al., 2010; Bulut, 2011; Kizilarslan and Ozhatay, 2012; Sagiroglu et al., 2012; Sarac et al., 2013; Akbulut and Ozkan, 2014; Korkmaz and Karakurt, 2015; Polat et al., 2015; Akbulut et al., 2017; Eminagaoglu et al., 2017; Gunes, 2017; Karci et al., 2017; Kartal and Gunes, 2017; Tuttu, 2017; Yesilyurt et al., 2017; Aydin and Yesil, 2018; Badem et al., 2018; Gurbuz et al., 2019; Karakose et al., 2019; Kazanci et al., 2020; Ergul Bozkurt, 2021; Guler et al., 2021; Gurdal and Ozturk, 2021; Kadioglu et al., 2021; Kazanci et al., 2021; Karakose, 2022a; Akbulut, 2022; Akbulut et al., 2022; Sener et al., 2022) have studied traditional medicine in Turkey’s northern Anatolia area (it extends through the Black Sea region to the Istranca Mountains in Thrace). These kinds of scientific research have also been carried out in several settlements of Kastamonu Province, the western Black Sea region where Taşköprü District is located (Sezik et al., 1992; Tuttu, 2017). However, apart from a questionnaire study about the uses of medicinal and aromatic plants in the region (Ozturk et al., 2017), there are no comprehensive scientific studies on traditional plant uses throughout the whole of Taşköprü District.

Our research thus aimed to record the remaining knowledge of folk medicine (primarily for humans, and if extant for animals) and to define the significance of medicinal plants for the inhabitants of the villages of Taşköprü District in Kastamonu.

We highlighted hitherto undocumented medicinal plant usages (Supplementary Table S1; Table 1) and documented new therapeutic usages in the region for any future studies of the area’s phytochemical or phytopharmacological characteristics. Along with any such ensuing studies, our study might show opportunities for regional economic development that will benefit the indigenous communities.

**TABLE 1:** The plants used in ethnoveterinary medicine in Taşköprü (Kastamonu/Turkey).

Botanical name, family and specimen number (new plant records for ethnoveterinary medicine in bold)	Local name (in Turkish)	Plant part used	Ailments treated/Therapeutic effect (new uses in bold)	Preparation	Administration, dosage	Rpt	CI	Literature uses
Amaryllidaceae								
*Allium sativum* L.^a^, MARE 19041	Sarımsak	Bulbils	Herb poisoning (in cattle-ovine)	Crushed and added into the ayran (a kind of drink made with yogurt and water)	Drunk (a bucket a day)	9	0.05	Herb poisoning (Tuzlaci and Tolon, (2000); Guler et al. (2021); Tuttu, (2017)), (Kultur, (2007); Guler et al. (2021))^b^
Betulaceae								
***Carpinus orientalis*** Mill., MARE 18166, 18372, 18952	Karaağaç	Stem bark	**Wound** (wolf, dog bite in animal)	Boiled	Ext. (wrapped in a cloth)	3	0.03	-
		Leaves	**Galactagogue** (in cattle-ovine)	−	Fed	2		
**C**upressaceae								
***Juniperus excelsa*** M. Bieb., MARE 18180, 18328	Ardıç, ömür ardıcı	Tar	**Wound** (in cattle, horse, donkey)	−	Ext	15	0.19	-
			**Scabies** (in cattle)	−	Ext	18		
			**Trichophytosis** (in ovine)	−	Ext	5		
*Juniperus oxycedrus* L., MARE 18143, 18205, 18283, 18319, 18860, 18895, 19032 [Syn.: *J. oxycedrus* L. subsp. *oxycedrus*]	Ardıç	Tar	Wound (in cattle-ovine, horse, donkey)	−	Ext	15	0.13	Wound (Guler et al. (2021))
			**Scabies** (in cattle)	−	Ext	6		
			**Trichophytosis** (in ovine)	−	Ext	5		
Fabaceae								
*Astracantha microcephala* (Willd.) Podlech, MARE 18165, 18267, 18968, 19053, 19087 [Syn.: *Astragalus microcephalus* Willd.]	Geven	Aerial parts	**Scabies** (in cattle-ovine)	Decoction	Ext	2	0.01	(Kazanci et al. (2021))^b^
***Astracantha microptera*** (Fisch.) Podlech, MARE 18307 [*Astragalus micropterus* Fisch.]	Geven	Aerial parts	**Increasing meat and milk yield** (in cattle-ovine)	Chopped	Fed	6	0.03	-
Fagaceae								
***Quercus infectoria*** subsp. ***veneris*** (A.Kern.) Meikle, MARE 18096, 18179, 18291, 18313, 18893, 18904 [Syn.: *Q. infectoria* subsp. *boissieri* (Reut.) O. Schwarz]	Kara meşe, meşe	Fruits	**Increasing meat and milk yield** (in cattle-ovine)	Crushed	Fed	2	0.01	-
*Quercus macranthera* subsp. *syspirensis* (K. Koch) Menitsky, MARE 18156	Meşe	Fruits	**Increasing meat and milk yield** (in cattle-ovine)	Crushed	Fed	2	0.01	(Kazanci et al. (2021))^b^
*Quercus petraea* subsp*. iberica* (Steven ex M.Bieb.) Krassiln*.*, MARE 18182, 19003, 19086	Boz meşe, meşe	Fruits	Increasing meat and milk yield (in cattle-ovine)	Crushed	Fed	2	0.01	Increasing meat and milk yield (Akbulut, (2022)), (Guler et al. (2021); Kazanci et al. (2021))^b^
Hypericaceae								
***Hypericum montbretii*** Spach, MARE 18330, 18374	Dağ çayı, kantaron, sarı çiçek, sarı ot	Flowering parts	**Trichophytosis** (in ovine)	Decoction	Ext	1	0.01	-
***Hypericum orientale*** L. Hypericaceae, MARE 18360	Dağ çayı, kantaron, sarı çiçek, sarı ot	Flowering parts	**Trichophytosis** (in ovine)	Decoction	Ext	1	0.01	-
*Hypericum perforatum* L. Hypericaceae, MARE 18251, 18308, 18369, 18378, 18415, 18963	Dağ çayı, kantaron, sarı çiçek, sarı ot	Flowering parts	**Trichophytosis** (in ovine)	Decoction	Ext	1	0.01	(Tuzlaci and Alparslan, (2007); Aydin and Yesil, (2018); Guler et al. (2021); Kazanci et al. (2021); Akbulut, (2022))^b^
Juglandaceae								
*Juglans regia* L.^a^, MARE 18162, 18322, 19037	Ceviz	Leaves	**Aphrodisiac** (in cattle)	Dried	Burned and smelled as incense	3	0.02	(Ezer and Mumcu Arisan, (2006); Bulut, (2011); Gurbuz et al. (2019); Guler et al. (2021); Akbulut, (2022))^b^
**Pinaceae**								
***Pinus nigra*** subsp**. *pallasiana*** (Lamb.) Holmboe, MARE 18155, 18221, 18293, 18320, 18928, 19048	Çam, kara çam	Resin	**Wound** (in cattle, horse, donkey)	Heated (mixed with wax and butter)	Ext	8	0.09	-
		Kindling	**Faciliator for seperation of placenta** (in cattle)	Bread is smoked with soot	Fed	1		
		Tar	**Scabies** (in cattle)	−	Ext	4		
			**Trichophytosis** (in ovine)	−	Ext	4		
*Pinus sylvestris* var. *hamata* Steven, MARE 18174, 18226, 18364, 18407, 18938	Çam, sarı çam	Resin	**Wound** (in cattle, horse, donkey)	Heated (mixed with wax and butter)	Ext	8	0.09	(Guler et al. (2021); Kazanci et al. (2021))^b^
		Kindling	**Faciliator for seperation of placenta** (in cattle)	Bread is smoked with soot	Fed	1		
		Tar	**Scabies**	−	Ext	4		
			**Trichophytosis**	−	Ext	4		
Ranunculaceae								
*Helleborus orientalis* Lam., MARE 18216, 18846, (2,6,8,10,15,16,26,40,47,48)^b^	Kesen otu	Aerial parts	Analgesic (in cattle)	−	Fed (a small amount)	3	0.03	Analgesic (Fujita et al. (1995); Gunes, (2017))
			**Postnatal vaginal discharge** (in cattle)	−	Fed (a small amount)	3		
Rosaceae								
*Prunus avium* (L.) L., MARE 18370, 18942, 18990 [Syn.: *Cerasus avium* (L.) Moench]	Kiraz, kuş kirazı	Fruit stalks	**Diuretic** (in cattle)	Decoction	Int. (a little bucket a day)	3	0.02	(Guler et al. (2021); Akbulut and Ozkan, (2014); Kazanci et al. (2021); Akbulut, (2022))^b^
Santalaceae								
*Viscum album* L., MARE 18115, 18159, 18849, 18929 [Syn.: *V. album* L. subsp. *album*]	Hurç, purç, ökse otu	Leafy branches	Increasing meat and milk yield (in cattle-ovine)	−	Fed	11	0.08	Increasing meat and milk yield Kultur, (2007); (Sarac et al. (2013); Gurbuz et al. (2019); Bussmann et al. (2020a); Kazanci et al. (2021))^b^
			**Strengthening** (in breeding cattle)	Boiled and mixed with flour	Fed	4		
***Viscum album*** L. subsp. ***austriacum*** (Wiesb.) Vollm., MARE 18454	Çam purcu, hurç, purç, ökse otu	Leafy branches	**Shortness of breath** (in hens)	Decoction	Int	2	0.09	-
			**Increasing meat and milk yield** (in cattle-ovine)	−	Fed	11		
			**Strengthening** (in breeding cattle)	Boiled and mixed with flour	Fed	4		

Rpt., Reports; Int., Internal use; Ext., External use; ^a^Cultivated plant. ^b^Different usage.

The aim of this study was:

1) Gathering and identifying plants that the local inhabitants use therapeutically in Taşköprü,
2) Recording information related to traditional folk medicine (both for humans and animals) and comparing this information with previous findings in northern Anatolia,
3) Evaluating both the cultural significance and the medicinal uses of the plant families and species in Taşköprü with cultural importance index (CI) and informant consensus factor (F_IC_) calculations.

## Materials and methods

### Study area

**FIGURE 1:**
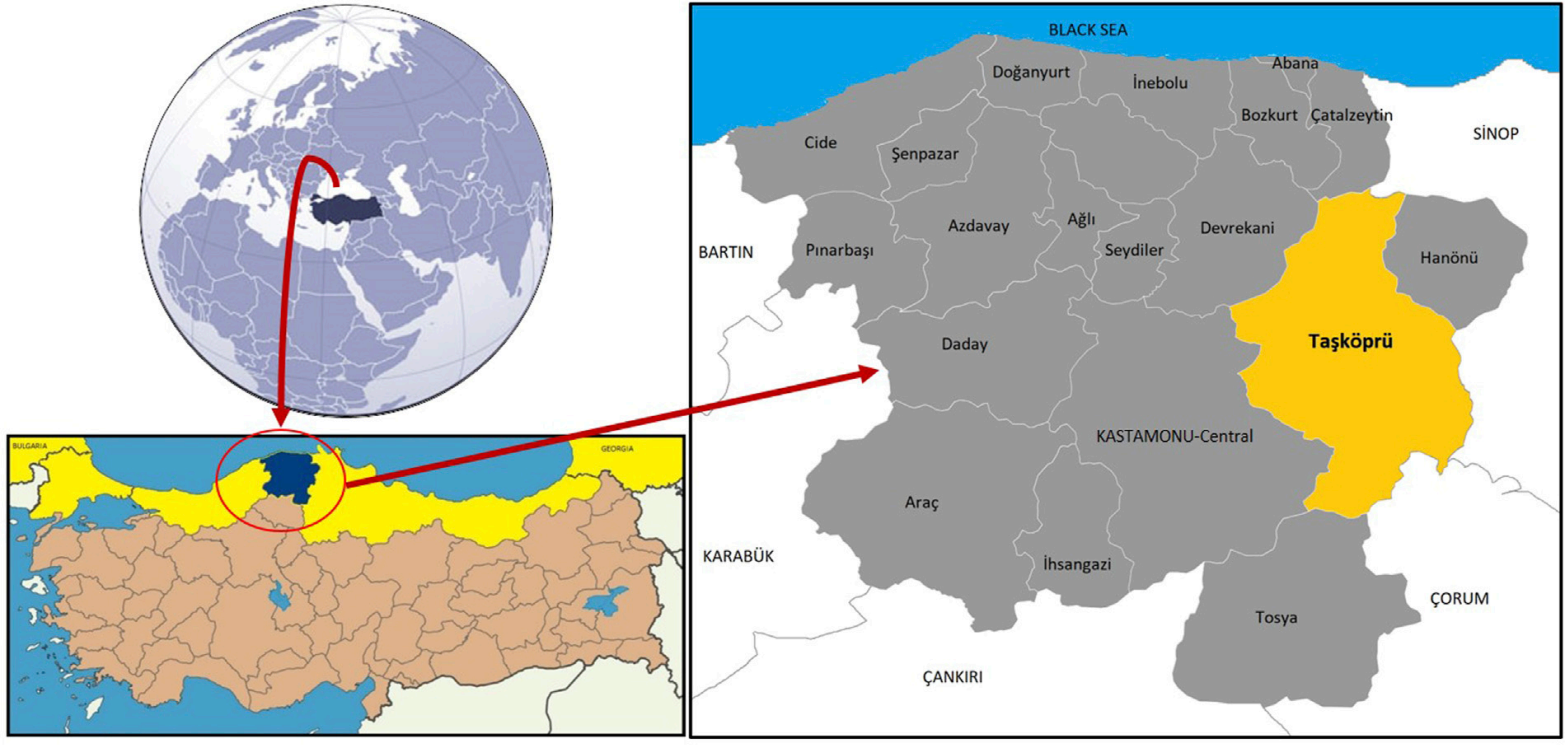
Geographical location of Taşköprü in Kastamonu-Turkey.

**FIGURE 2:**
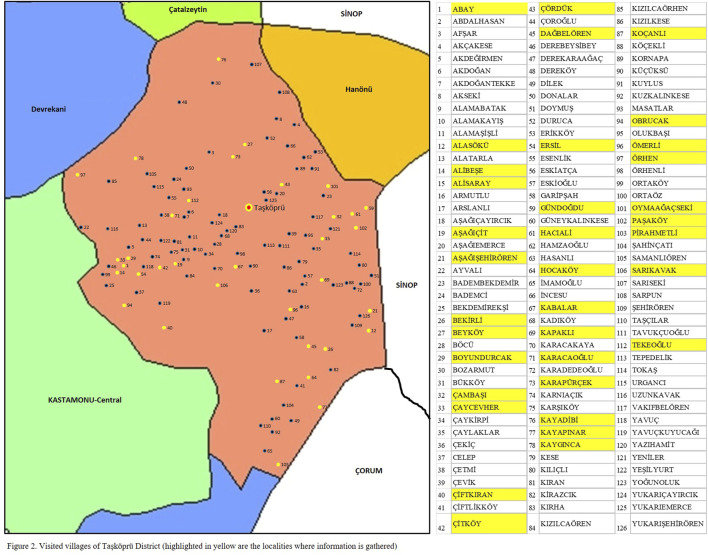
Visited villages of Taşköprü District (highlighted in yellow are the localities where information is gathered).

Taşköprü District is located (41⁰10′30″−41⁰45′31″ N, 33⁰54′50″−34⁰28′33″ E) in Kastamonu Province in Turkey’s western Black Sea region at an altitude of 550 m. Taşköprü is 1,811 km^2^, and is bordered by Boyabat (Sinop) in the east, Hanönü (Kastamonu) in the northeast, Türkeli (Sinop) and Çatalzeytin (Kastamonu) in the north, Devrekani (Kastamonu) in the northwest, Kastamonu-Central district in the west, Tosya (Kastamonu) in the south and Kargı (Çorum) in the southeast (Figure 1). Taşköprü has a population of 37,439 and consists of one town and 126 villages (Figure 2). The town contains 16,851 people, and the other 20,588 people live in the countryside (TUIK, 2022).

The language of the region is Turkish. Taşköprü District is one of Turkey’s most popular places, mainly because of its famous 14th-century stone bridge (“Taşköprü”) over the Gök River, the remains of the ancient city of Pompeiopolis, and Taşköprü garlic (known as “white gold”). This garlic, with its unique flavour, is the district’s most prominent agricultural product, followed by beet, barley and wheat. The economic structure of Taşköprü is based on agricultural industry, forestry and animal husbandry (KUZKA, 2022).

The Küre Mountains–a notable area of plants in Turkey–are located in the north of the district, and an extension of the Ilgaz Mountains is located in the south (Ozhatay et al., 2005). For this reason, the north and south of the district are bordered by rich forest regions which include important plant areas. Previous floristic studies in this region recorded 283 plant taxa from around the Ilgaz Mountains (Pehlivan, 2007), 277 plant taxa from around the Küre Mountains (Demirbas Ozen et al., 2013) and 374 plant taxa from around Mount Yaralıgöz (Karakose and Terzioglu, 2019). Mount Çangal and Mount Elek, north and east of the town of Taşköprü, are the district’s highest mountains. Their average elevation exceeds 1,500 m. The Gök River is the district’s most important river, due to the plain formed around it (Directorate of Culture and Tourism, 2022).

The region’s climate is typical Black Sea: the annual average temperature is 18°C and the annual average rainfall is 604.9 mm (Goverment Bureau, 2022).

**FIGURE 3:**
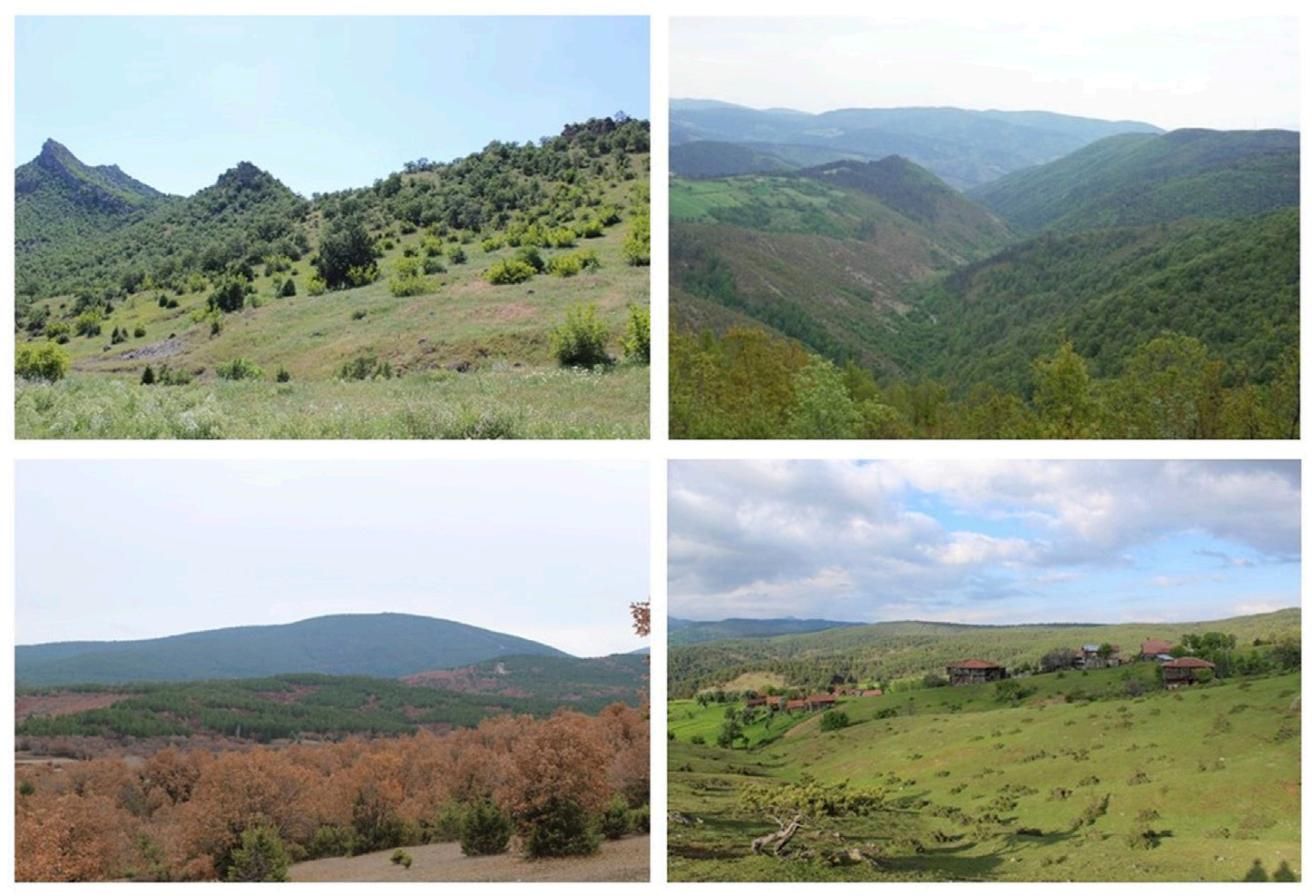
General views from the vegetation.

This phytogeographical area is a part of what is known as the Circumboreal Region, and it is referred to as the Euxine section of Anatolia. Covering the bulk of Georgia and the Caucasus, it extends through most of northern Anatolia to the Istranca Mountains in Thrace and southeast Bulgaria. Forest and shrub cover the majority of the area below the tree line, and this cover is generally deciduous trees and evergreen shrubs in the lower areas. However, conifers increase and even predominate in the higher parts (Davis, 1965-1985) (Figure 3).

The characteristic elements of this region’s slopes and clearings in the study area are mainly trees and shrubs: *Abies nordmanniana* subsp. *equi-trojani* (Asch. et Sint. ex Boiss.) Coode et Cullen, *Juniperus excelsa* M. Bieb., *J. oxycedrus* L., *Pinus nigra* subsp. *pallasiana* (Lamb.) Holmboe, *P*. *sylvestris* var. *hamata* Steven, *Fagus orientalis* Lipsky, *Carpinus orientalis* Mill., *Quercus infectoria* subsp. *veneris* (A.Kern.) Meikle, *Quercus petraea* subsp. *iberica* (Steven ex M.Bieb.) Krassiln., *Tilia rubra* subsp. *caucasica* (Rupr.) V. Engl., *Acer campestre* L., *Corylus avellana* L., *Cornus mas* L., *C. sanguinea* subsp. *australis* (C.A.Mey.) Jáv., *Sambucus nigra* L., *Crataegus* spp., *Rhododendron luteum* Sweet, and *Cistus laurifolius* L.. The most common and most frequently encountered herbs observed and recorded in the field were *Helleborus orientalis* Lam., *Chelidonium majus* L., *Euphorbia seguieriana* Neck., *Salvia sclarea* L., *Sambucus ebulus* L., *Petasites hybridus* (L.) G.Gaertn., B.Mey. et Scherb., *Centranthus longiflorus* Steven and *Valeriana alliariifolia* Vahl..

Additionally, Kastamonu and its environs (including Taşköprü) are important areas for the production of sahlep-tuber (Karakose and Terzioglu, 2019).

### Data collection

This study was conducted following the guidelines for best practices in ethnopharmacological research (Heinrich et al., 2018; Weckerle et al., 2018; Leonti 2022). Our main purpose was to gather information about medicinal plants used by the informants. If available, information about plants used for animal health was also collected. Firstly, when possible, “mukhtars” (village headmen) or prominent villagers were consulted to identify people who were interested and experienced in herbal treatments. When not possible, these interested and experienced people were identified by villagers we chanced to meet. In addition, since the hometown of the wife of one of the researchers is in the research area, an atmosphere of trust between the two parties was quickly and easily established in each village visited. The information was obtained through semi-structured interviews within the framework of an open-ended questionnaire (Supplementary Table S2). In order to allow the participants to speak freely and spontaneously, the interviewers did not articulate any guidelines for the items of discussion; they simply bore the guidelines in mind. These questions also appear in our previous work (Emre et al., 2021).

Taşköprü, with its central city and 126 villages, was visited on various occasions in different seasons and months between 2016 and 2018, and the field work was carried out over a total of 71 days. From all the locations visited (Figure 2), information was collected in the central city and 35 villages (highlighted in yellow in Figure 2).

During our conversations, a total of 197 people were interviewed. The geographical distribution of informants in the research area was 13 from the central city (6.6%) and 184 from the villages (93.4%). The gender distribution of informants was 109 men and 88 women. The range of their ages was from 32 to 79, with an average age of 53. Mainly patients and experienced adults provided the information and data: the local names, parts and therapeutic effects of plants used, the disorders treated and the methods plants were prepared or administered. The interviewees’ occupations were farmer, housewife, shepherd, labourer (forestry and related industries) and mukhtar. Interviews were conducted in various places (house, garden, woodland, field, etc.).

The collection of plant vouchers was usually done with the informants. Specimens were sometimes collected first, with the interviews following.

Informed consent was obtained orally before each interview, and the Code of Ethics of the International Society of Ethnobiology (ISE, 2008) was strictly followed.

Identifying the plant samples collected was made with “The Flora of Turkey and East Aegean Islands” (Davis, 1965-1985; Davis et al., 1988; Guner et al., 2000) and “Illustrated Flora of Turkey Vol 2” (Guner et al., 2018). The scientific names of plant taxa were checked using the Turkish Plant List (Guner et al., 2012) and updated according to World Flora Online (WFO, 2021). The threat categories of some plant taxa were determined according to Ekim et al. (2000) and to the International Union for Conservation of Nature (IUCN) (2021).

Herbarium samples were stored at the Herbarium of the Faculty of Pharmacy at Marmara University (MARE). The identification of three taxa (*Allium cepa, Beta vulgaris, Citrullus lanatus*) without herbarium samples was made in line with our personal observations in the field, and this is shown in “Supplementary Table S1” as “Obs.”

### Calculations

Ethnobotanical data were collected from 197 local informants. The statistical analysis structure in use-reports (UR) included the cultural importance index (CI) for every taxon (Supplementary Table S1, Table 1) and the informant consensus factor (F_IC_) to evaluate the data (Table 2).

**TABLE 2:** F_IC_ Values of category of ailments.

Ailment categories	Number of use report (n*ur*)	Number of taxa (n*t*)	Informant consensus factor (F_IC_)
Respiratory system	973	49	0.95
Skin and subcutaneous tissue	625	38	0.94
Nervous system	63	6	0.92
Circulatory system	321	41	0.88
Bones, jointsetc.	101	15	0.86
Digestive system	157	38	0.76
Genital–urinary system	104	26	0.75
Ethnoveterinary uses	173	19	0.89

The Cultural Importance Index (CI) (Tardío and Pardo-de-Santayana, 2008) comparatively measures the significance of commonly-used species based on the perceptions of informants. The CI was calculated with the formula *CI = URs/N*; *UR* (Use Report) = the total number of recorded uses for each species; *N* = the total number of the study’s informants.

Another quantitative method, the informant consensus factor gives the relationship between the number of use-report in each category-was calculated using the formula F_IC_ *=* (*Nur—Nt*)*/*(*Nur—1*). *Nur* is the number of citations used in each category; *Nt* is the number of species used. Such a process shows data homogeneity. When informants randomly choose plants or do not supply information regarding plant uses, the F_IC_ is near zero. When the community has well-defined selection criteria, and/or if the informants supply information between their values, the F_IC_ is near one (Trotter and Logan, 1986; Heinrich et al., 1998). Plants with a higher F_IC_ value are thought more likely to effectively treat a particular ailment (Heinrich, 2000).

F_IC_ values were created for eight medicinal use-categories (including ethnoveterinary) and for several emic subcategories, all of which were arranged (Table 2) according to the International Classification of Primary Care (ICPC-2) (WICC, 1998).

## Results

### Demographic characteristics of informants

**FIGURE 4:**
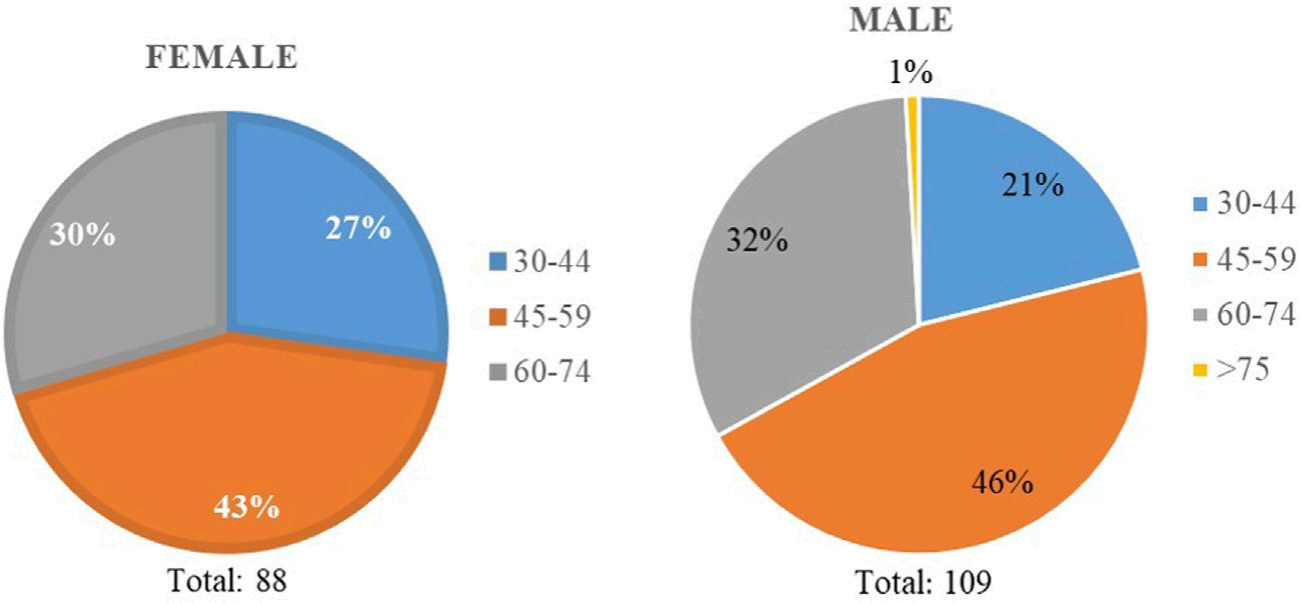
Age groups of informants.

The informants’ demographic traits, recorded during the in-person semi-structured interviews, are given in Table 3. There were 197 interviewees: 44.7% of them were female and 55.3% were male. The ages of the informants ranged from 30 to over 75: 23.9% were 30–44 years old, 44.6% were 45–59 years old, 31% were 60–74 years old, and 0.5% were over the age of 75 (Figure 4). All the informants were native to Taşköprü District: 93.4% were village dwellers and 6.6% town dwellers. Furthermore, 96.5% of them were literate. Occupationally, one-third of the informants (34.5%) were housewives.

**TABLE 3:** Demographic characteristics of the informants (*n* = 197).

	Features	Number	Frequency (%)
Gender	Female	88	44.7
	Male	109	55.3
Educational level	Illiterate	7	3.5
	Primary school	99	50.3
	Secondary school	56	28.4
	High school	33	16.8
	University	2	1.0
Age groups	30–44	47	23.9
	45–59	88	44.6
	60–74	61	31.0
	˃75	1	0.5
Occupation	Housewife	68	34.5
	Farmer	55	27.9
	Shepherd	19	9.7
	Worker	23	11.7
	Artisan	4	2.0
	Retired	28	14.2
Place, where lived	Town	13	6.6
	Village	184	93.4

### Medicinal plants and associated knowledge

**FIGURE 5:**
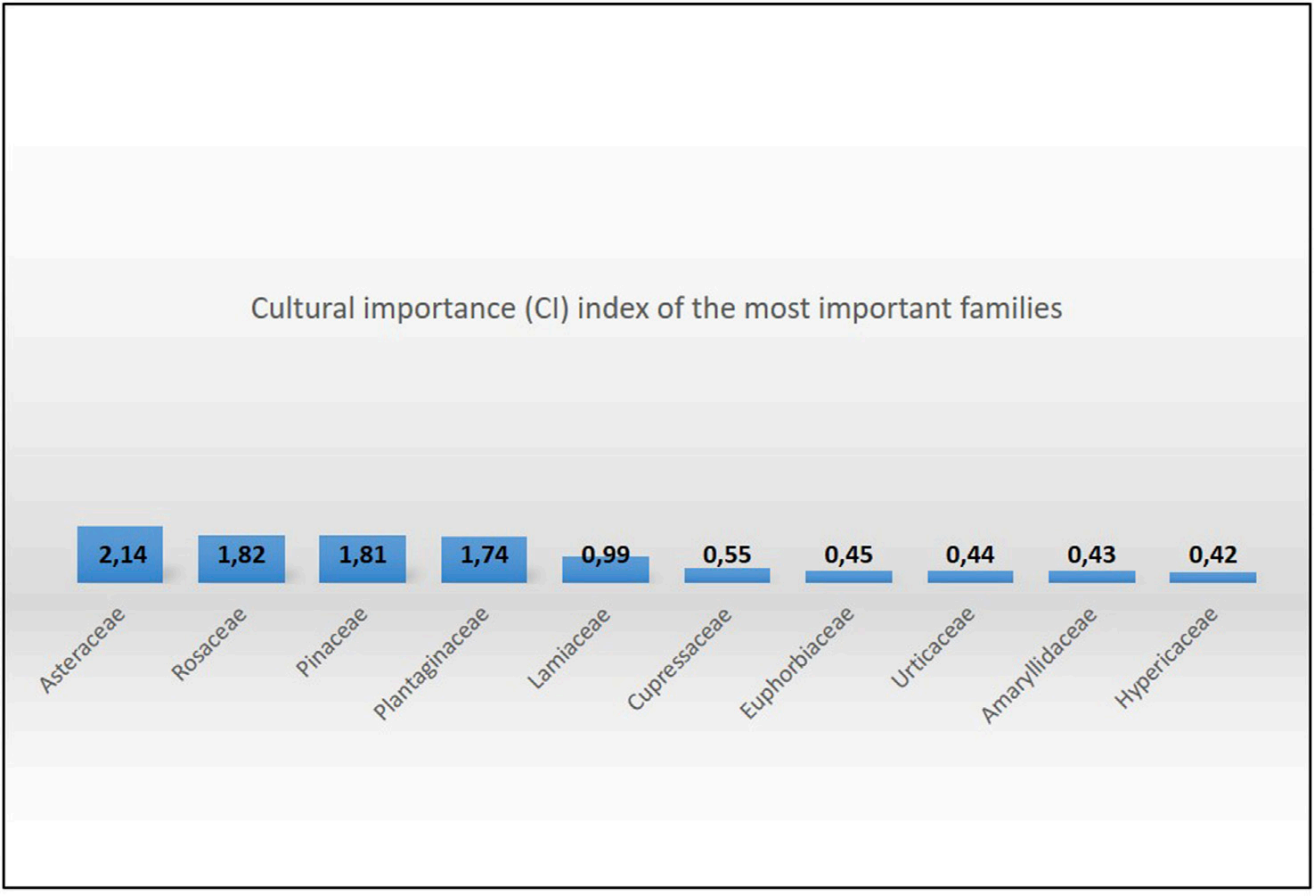
Cultural importance (CI) index of the 10 most important families in Taşköprü.

The folk medicine plants utilized in Taşköprü are in Supplementary Table S1, Table 1. They are listed in alphabetical order by family and botanical names, and with their related data. Taxonomic changes are shown in parenthesis in Supplementary Table S1, Table 1 along with any popular scientific names. In the course of this research, 264 specimens were gathered and 101 medicinal taxa from 31 families were identified. Of these, 89 species were wild and 12 were cultivated. Cultural importance (CI) index of the most prevalent families were Asteraceae (2.14), Rosaceae (1.82), Pinaceae (1.81), Plantaginaceae (1.74) and Lamiaceae (0.99) (Figure 5).

**FIGURE 6:**
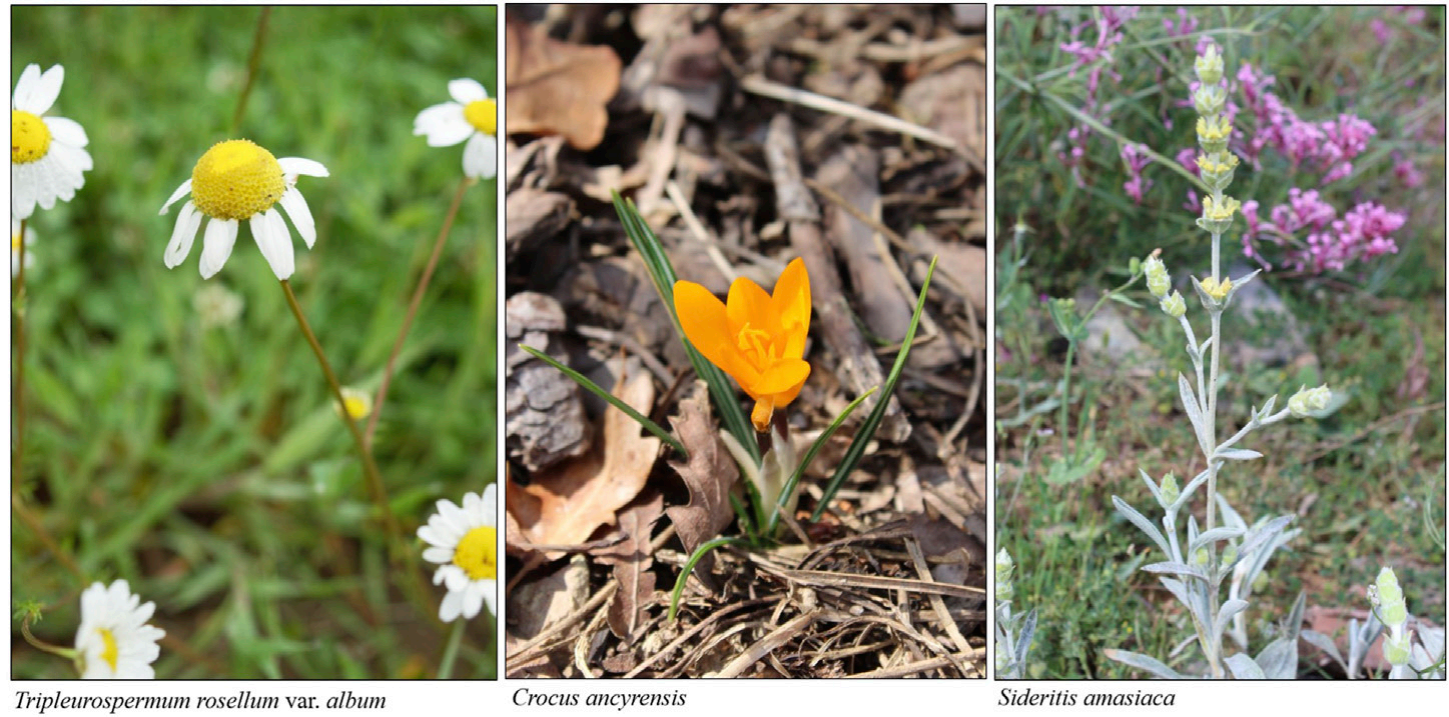
A view of the endemics in the research area.

There are some endemics of Turkey among the plants with medicinal use in Taşköprü: *Abies nordmanniana* subsp. *equi-trojani* (Asch. et Sint. ex Boiss.) Coode et Cullen, *Anthemis sintenisii* Freyn, *Astracantha microptera* (Fisch.) Podlech, *Crataegus tanacetifolia* (Poir.) Pers., *Crocus ancyrensis* (Herb.) Maw, *Helichrysum aucheri* Boiss., *Quercus macranthera* subsp. *syspirensis* (K. Koch) Menitsky, *Sideritis amasiaca* Bornm. and *Tripleurospermum rosellum* (Boiss. et Orph.) Hayek var. *album* E. Hossain (Davis, 1965-1985; Davis et al., 1988; Guner et al., 2000; Guner et al., 2012) (Figure 6). All these endemics are listed as least concern (LC) with the exceptions of *Sideritis amasiaca* and *Tripleurospermum rosellum* var. *album*, which are listed vulnerable (VU) according to Ekim et al. (2000) and the IUCN Red List (2021).

### Plant parts and preparation methods

**FIGURE 7:**
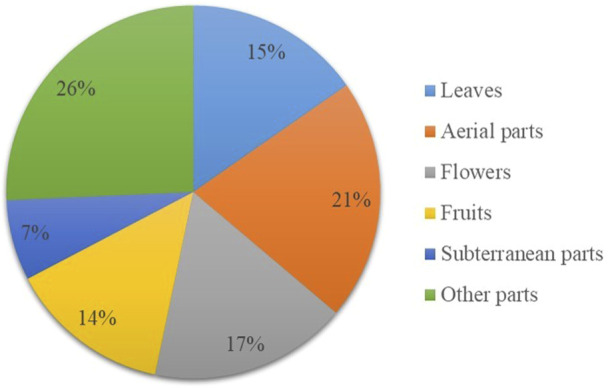
Used parts of the medicinal plants in the study.

The plant parts used to treat different ailments were aerial (21%), flowers (17%), leaves (15%), fruits (14%), subterranean (7%) and other (26%) (Figure 7). The locals occasionally used ingredients like olive oil, lemon/juice, sugar, honey, wax, butter, milk or flour when preparing remedies.

The preparation methods were decoction (38.4%), direct application (24.1%; no preparation whatsoever), infusion (11.8%), crushing (4.4%), molasses (3.6%) and other (17.7%; oleate, powdering, roasting, heating, boiling, etc.).

Since the diseases seen in the region are mainly respiratory system, skin and subcutaneous tissue, nervous system and circulatory system disorders, it is seen that plants are mostly used internally in the form of medicinal tea or externally in various forms in the treatment of these diseases. Therefore, it can be thought that a small number of plants are used as food-medicine.

The study recorded 499 remedies, and most were for internal application (62.3%) (Supplementary Table S1, Table 1).

Medicinal plants used in multi-herbal recipes with more than one species are listed in Table 4. Three of them, *Cerasus vulgaris* Mill., *Citrus limon* (L.) Burnm. fil. and *Olea europaea* L. var. *europaea,* were used only in multi-herbal recipes.

**TABLE 4:** Multiherbal recipes used as folk medicine in Taşköprü (Kastamonu/TURKEY).

Recipe	Plant	Plant part used	Ailments treated, therapeutic effect	Preparation	Adm	Use report
1	*Allium cepa, Allium sativum*	Bulbs, Bulbils	Cough, Expectorant (in infants)	Grated, then filtered and juice mixed with honey	Int	6
2	*Cydonia oblonga, Tilia rubra* subsp. c*aucasica, Rosa canina, Malus sylvestris*	Leaves, Flowers, Fruits, Exocarp	Cough	Decoction	Int	3
3	*Rosa canina, Tilia rubra* subsp. c*aucasica, Thymus longicaulis* subsp. *longicaulis*/*T. praecox*	Fruits, Flowers, Aerial parts	Cold	Decoction	Int	3
4	*Rosa canina, Tilia rubra* subsp. c*aucasica, Citrus limon*	Fruits, Flowers, Fruit juice	Cold	Decoction	Int	2
5	*Urtica dioica*/*U. urens, Cerasus vulgaris*	Aerial parts, Fruit stalks	Diuretic	Decoction	Int	2
6	*Matricaria chamomilla, Thymus longicaulis* subsp. *longicaulis*	Capitula, Aerial parts	Sore throat	Decoction	Int	4
7	*Allium cepa, Olea europaea* var. *europaea*	Bulbs, Pericarp	Bruise	Crushed and mixed	Ext	3

According to the informants, two of the medicinal plants–*Onopordum acanthium* L. and *O. tauricum* Willd.–were not used anymore because malaria was no longer seen in Taşköprü.

*Malva neglecta* Wallr. and *Malva sylvestris* L. species are no longer used in the study area for abortion because they cause gynecological disorders such as excessive bleeding. In addition, some sources in the literature state that these species are used to similar ends (Kultur, 2007; Tuttu, 2017; Kazanci et al., 2020).

### Plant names

Recording the local names of plants in terms of folk culture about plants helps to get an idea about the recognition of plants. Such ethnobotanical studies we have done also constitute a source for these records In many instances, the locals used the same vernacular name to refer to two or more different plant species. These species are presented in Table 5. We see there that in some cases different species of the same genus have the same names, and that in other cases species of different genera have the same names.

**TABLE 5:** The same vernacular name(s) used for more than one plant species.

Local name(s)	Botanical name(s)
Sahlep, salep	*Anacamptis pyramidalis–Dactylorhiza romana* subsp. *romana–D. uvilleana–Orchis coriophora*—*O. morio* subsp. *morio—O. purpurea–O. simia–O. tridentata*
Papatya	*Anthemis cotula–A. sintenisii–Cota tinctoria* var. *pallida–Matricaria chamomilla–Tripleurospermum rosellum* var. *album*
Geven	*Astracantha microcephala–A. microptera*
Alıç, öküzgötü, yemişen	*Crataegus azarolus* var. *pontica–C. monogyna–C. orientalis* subsp. *orientalis–C. rhipidophylla* var. *rhipidophylla–C. tanacetifolia*
Kındıra, ulasır otu, kırkkilit otu, beygir otu	*Equisetum arvense–E.* p*alustre–E. telmateia*
Sütlü ot	*Euphorbia esula* subsp. *tommasiniana–E. seguieriana* subsp. *niciciana–E. seguieriana* subsp. *seguieriana–Lactuca serriola*
Dağ çayı, kantaron, sarı çiçek, sarı ot	*Hypericum montbretii–H. orientale–H. perforatum*
Ardıç	*Juniperus excelsa–J. oxycedrus*
Ebegümeci, ebegömeci, ebemgümeci	*Malva neglecta–Malva sylvestris*
Bertik otu	*Marrubium anisodon–M. vulgare*
Diken otu	*Onopordum acanthium–O. tauricum*
Gelincik	*Papaver dubium–P. rhoeas*
Kabalak	*Petasites hybridus–Salvia sclarea*
Çam	*Pinus nigra* subsp. *pallasiana–P. sylvestris* var. *hamata*
Damar otu, siğil otu, siğil yaprağı, sinir otu, sinir yaprağı	*Plantago lanceolata–P. major* subsp. *intermedia–P. major* subsp. *major*
Meşe	*Quercus infectoria* subsp. *veneris–Q. macranthera* subsp. *syspirensis–Q. petraea* subsp*. iberica*
Böğürtlen, kır böğürtleni	*Rubus canescens* var. *canescens–R. canescens* var. *glabratus–R. hirtus–R. sanctus*
Söğüt	*Salix alba–S.* x *fragilis*
Adaçayı	*Salvia tomentosa–S. verticillata* subsp. *amasiaca–Sideritis amasiaca*
Kekik	*Teucrium polium–Thymus longicaulis* subsp*. longicaulis–T. praecox*
Isırgan	*Urtica dioica–U. Urens*
Hurç, purç, ökse otu	*Viscum album* subsp. *album–V. album* subsp. *austriacum*

### Data analyses

The reports obtained were separated into 8 categories of use which grouped the illnesses into ethnomedicinal categories that were based on the emic perceptions of the informants (Table 2) according to the ICPC-2.

Four of the above-mentioned categories of medicinal use are represented by the most common plant species with high UR in these categories. They are ranked by the number of URs for each use category in Table 6.

**TABLE 6:** The most common plant species with high UR in medicinal categories.

Ailment categories	Botanical name	Number of use report (N*ur*)
Respiratory system		
	*Pinus nigra* subsp. *pallasiana*	116
	*Pinus sylvestris* var. *hamata*	113
	*Anthemis cotula*	55
	*Anthemis sintenisii*	55
	*Cota tinctoria* var. *pallida*	55
	*Cydonia oblonga*	55
	*Matricaria chamomilla*	55
	*Tripleurospermum rosellum* var. *album*	55
	*Rosa canina*	52
Skin and subcutaneous tissue		
	*Plantago lanceolata*	102
	*Plantago major* subsp. *intermedia*	102
	*Plantago major* subsp. *major*	102
	*Euphorbia esula* subsp. *tommasiniana*	30
	*Euphorbia seguieriana* subsp. *niciciana*	30
	*Euphorbia seguieriana* subsp. *seguieriana*	30
	*Juniperus excelsa*	24
Nervous system		
	*Anthemis cotula*	12
	*Anthemis sintenisii*	12
	*Cota tinctoria* var. *pallida*	12
	*Matricaria chamomilla*	12
	*Tripleurospermum rosellum* var. *album*	12
Ethnoveterinary uses		
	*Juniperus excelsa*	38
	*Juniperus oxycedrus*	26
	*Pinus nigra* subsp. *pallasiana*	17
	*Pinus sylvestris* var. *hamata*	17
	*Viscum album* subsp. *austriacum*	17

The main ailments, based on the URs, were wounds (UR: 404), the common cold (UR: 298), coughs (UR: 277), shortness of breath (UR: 270), diabetes (UR: 161), cardiovascular system diseases (UR: 70), rheumatism (UR: 64), sedative (UR: 63), warts (UR: 55), haemorrhoids (UR: 55), abdominal pain (UR: 35), bronchitis (UR: 32), constipation (UR: 28) and urinary system diseases (UR: 24) (Supplementary Table S1, Table 1).

According to the F_IC_ numbers, respiratory system complaints (mainly the common cold, coughs and shortness of breath) had the highest degree of consensus at 0.95 (Table 2). This study found that approximately 50% (49 plant) of all recorded taxa were used to treat various respiratory system disorders (URs: 973). Moreover, *Pinus nigra* subsp. *pallasiana* (URs: 116), *Pinus sylvestris* var. *hamata* (URs: 113), *Anthemis cotula*, *A. sintenisii, Cota tinctoria* var*. pallida, Cydonia oblonga, Matricaria chamomilla, Tripleurospermum rosellum* var*. album* (URs: 55 each) and *Rosa canina* (URs: 52) are ranked in accordance with the highest number of URs for respiratory diseases. In particular, *Rosa canina* (URs: 36), *Cydonia oblonga* (URs: 40) and *Pinus sylvestris* var. *hamata* (URs: 59) are the most important species used for the common cold, coughs, and shortness of breath, respectively. The most frequently mentioned therapy consists of mainly infusion and decoction of plant parts. These are a useful form of treatment often used for respiratory diseases. Besides, the inner layer of the stem bark, known as “soymuk” obtained from *Abies nordmanniana* subsp. *equi-trojani*, *Pinus nigra* subsp. *pallasiana* and *P. sylvestris* var. *hamata* is commonly used in the treatment of shortness of breath. It is widely used for this purpose in the villages of Taşköprü, and was also reported by Fujita et al. (1995) and Tuttu (2017).

These findings were not unexpected: the local inhabitants engage in activities such as agriculture, animal husbandry and forestry in all seasons and under difficult geographical conditions in order to earn a living.

Next were skin and subcutaneous tissue ailments (mainly wounds and warts) at 0.94 (Table 2). This study identified that nearly 40% (38 plant) of all recorded taxa were used to treat skin and subcutaneous tissue disorders and 625 of the use reports fall into this category. In addition, *Plantago* spp. (URs: 102 each), *Euphorbia* spp. (URs: 30 each) and *Juniperus excelsa* (URs: 24) are ranked in accordance with the highest number of URs for skin and subcutaneous tissue disorders. Of these, leaves of *Plantago* spp. (URs: 97 each) and latex of *Euphorbia* spp. (URs: 18 each) are the most common plant parts applied to the wounds and warts, respectively. Dermatological disorders are mostly treated topically with directly use of plant parts, poultice and oleate in Taşköprü. It is seen that the poultice obtained from the aerial parts of *Euphorbia* spp. is prepared by boiling with milk, unlike other preparation methods in this category.

Carrying out agricultural, animal husbandry and forestry activities without protective measures and in a harsh climate is thought to be the common cause of skin diseases in the region.

The third highest degree of consensus was nervous system disorders (mainly sedative) at 0.92 (Table 2). This study identified six plant taxa (URs: 63) used for nervous system disorders. *Anthemis cotula*, *A. sintenisii, Cota tinctoria* var*. pallida, Matricaria chamomilla* and *Tripleurospermum rosellum* var*. album* (URs: 12 each), locally called “papatya = chamomile” are the most important species used as a sedative. The most frequently mentioned therapy consists of mainly infusion of plant parts.

Again, the reason for these findings was the local inhabitants earning a livelihood under stressful and difficult conditions.

The fourth highest, according to the F_IC_ number (0.89), was the ethnoveterinary uses category. In addition to human diseases, it was found that 19 plant taxa (URs: 173) were used to treat animal diseases. *Juniperus excelsa* (URs: 38), *Juniperus oxycedrus* (URs: 26), *Pinus* spp. and *Viscum album* subsp. *austriacum* (URs: 17 each) are ranked in accordance with the highest number of URs for ethnoveterinary diseases. In particular, *Juniperus excelsa* (URs: 18), *Juniperus* spp (URs: 15 each) and *Viscum* spp. (URs: 11 each) are the most important species used for the scabies, wound, and increasing meat-milk yield, respectively. Among the parts used, tar obtained from *Juniperus* spp. and *Pinus* spp. is directly applied on wounds and scabies for healing.

These cases are likely the result of injuries, exposure to parasites, or delayed treatment of animals grazing in rugged and widespread areas - especially in the spring.

These four groups are followed by circulatory system (0.88), bones, joints, etc. (0.86), the digestive system (0.76) and genital–urinary system (0.75) (Table 2).

## Discussion

### Comparison with previous studies

Some of the plants in Supplementary Table S1, Tables 1, 4 are well-known in Turkey and recorded previously in numerous ethnobotanical researches carried out in various areas of northern Anatolia (Sezik et al., 1991; Sezik et al., 1992; Fujita et al., 1995; Yazıcıoglu and Tuzlaci, 1996; Sezik et al., 1997; Yesilada et al., 1999; Tuzlaci and Tolon, 2000; Uzun et al., 2004; Ecevit Genc and Ozhatay 2006; Ezer and Mumcu Arisan, 2006; Turkan et al., 2006; Cansaran et al., 2007; Kultur, 2007; Tuzlaci and Alparslan, 2007; Akgul, 2008; Koyuncu et al., 2009; Koca and Yildirimli, 2010; Tuzlaci et al., 2010; Bulut, 2011; Kizilarslan and Ozhatay, 2012; Sagiroglu et al., 2012; Sarac et al., 2013; Akbulut and Ozkan, 2014; Korkmaz and Karakurt, 2015; Polat et al., 2015; Akbulut et al., 2017; Eminagaoglu et al., 2017; Gunes, 2017; Karci et al., 2017; Kartal and Gunes, 2017; Tuttu, 2017; Yesilyurt et al., 2017; Aydin and Yesil, 2018; Badem et al., 2018; Gurbuz et al., 2019; Karakose et al., 2019; Kazanci et al., 2020; Ergul Bozkurt, 2021; Guler et al., 2021; Gurdal and Ozturk, 2021; Kadioglu et al., 2021; Kazanci et al., 2021; Karakose, 2022a; Akbulut, 2022; Akbulut et al., 2022; Sener et al., 2022) found that *Plantago major* subsp. *major* was the most widely used medicinal plant and was recorded at 33 locations around Taşköprü in North Anatolia. Comparisons of our findings and earlier findings are in Supplementary Table S1, Table 1.

These studies mentioned above also found that the widely-distributed species *Hypericum perforatum*, *Juglans regia*, *Cydonia oblonga, Rosa canina, Malva sylvestris* and *Cerasus avium* were the main plants used in folk remedies over the region.

**FIGURE 8:**
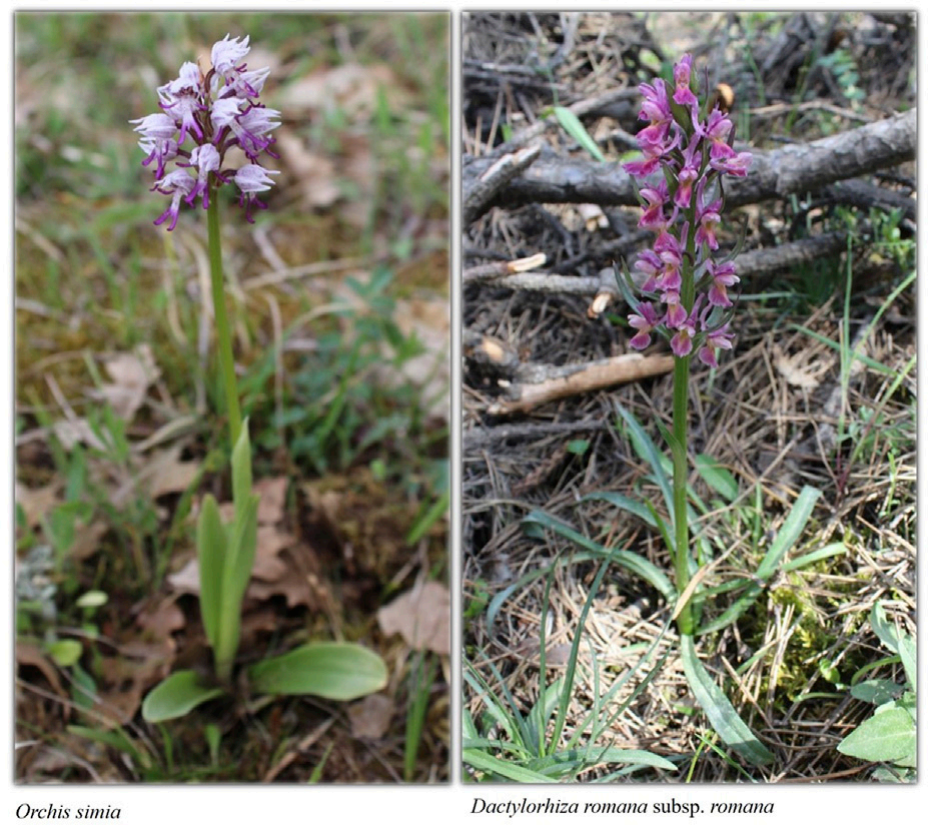
A view of the widespread orchids from the region.

We were informed that the local inhabitants used to collect and sell *Hypericum perforatum* to supplement their income, but this practice was short-lived. We were also informed that species of *Anacamptis, Dactylorhiza* and *Orchis* genera were used as traditional folk medicine in the region, and that their tubers have long been harvested for income (Figure 8). As has already been reported, these plants are under threat due to their collection for commercial purposes (Ozhatay et al., 2005). It is also seen that *Anacamptis pyramidalis*, *Dactylorhiza romana* subsp*. romana*, *D. urvilleana*, *Orchis coriophora, O. purpurea*, *O. simia*, *O. tridentata* are listed as least concern (LC) and *Orchis morio* subsp. *morio* is listed as near threatened (NT) according to “The IUCN Red List (2021).” These taxa are also protected by the CITES, 2013 (Convention on International Trade in Endangered Species of Wild Fauna and Flora). In this regard, we used every opportunity to urge the local inhabitants not to over-collect wild orchids, especially for economic or medical purposes.

Besides verifying previously gathered data from the region, this study in Taşköprü in northern Anatolia recorded for both humans and animals a total of 303 new therapeutic uses of 101 plant taxa. In addition, 20 of these 101 taxa were recorded for the first time (new plant records and new uses are indicated in bold in Supplementary Table S1, Table 1) by this study.

Furthermore, this study recorded a total of 29 new therapeutic uses of 19 plant taxa in the field of ethnoveterinary medicine. Eight of these taxa (*Astracantha microptera, Carpinus orientalis, Juniperus excelsa, Quercus infectoria* subsp*. veneris, Hypericum montbretii, Hypericum orientale, Pinus nigra* subsp*. pallasiana* and *Viscum album* subsp*. austriacum*) were recorded for the first time as ethnoveterinary medicinal plants.

Also, two of the 19 ethnoveterinary medicinal plants (*Astracantha microptera* (Fisch.) Podlech and *Helleborus orientalis*) were used only for animal health, while the other 17 were used for both humans and animals in Taşköprü (Table 1).

Moreover, our findings show similarities to and differences from previously recorded uses of all the ethnoveterinary medicinal plants mentioned above and in the references (Table 1).

Consistent with the reports of this study, the most frequently quoted species in the literature are: *Helleborus orientalis* (10), *Hypericum perforatum*, *Juglans regia*, *Viscum album* subsp. *album* (5 each) and *Allium sativum, Prunus avium* (4 each).

The uses of *Allium sativum* bulbils in the treatment of plant poisoning, the tar of *Juniperus oxycedrus* in the treatment of wounds, the aerial parts of *Helleborus orientalis* as an analgesics and the fruits of *Quercus petraea* subsp. *iberica* and the leafy branches of *Viscum album* in increasing meat and milk yield are similar. Regarding the plant parts, aerial parts (with leaves, fruits, flowers) were the commonly used part in Taşköprü. It is followed by tar, resin and kindling. The remedies were applied mainly externally and the most common preparation method was direct application of plant parts (Table 1).

It can be said that there are two reasons for the abundance of data about ethnoveterinary medicine: almost every house in the region engages in animal husbandry to provide both income and food, and veterinary services are expensive and difficult to access. In recent years, however, it has been observed that animal husbandry in the villages is decreasing due to urban migration and to rising costs and diminishing profits. It is obvious that this knowledge will eventually be forgotten.

We also compared our ethnomedicinal data with data previously gathered from the Balkans to the Caucasus, as we did for our data and previous findings in northern Anatolia (Ivancheva and Stantcheva, 2000; Koleva et al., 2015; Bussmann et al., 2016a; Bussmann et al., 2016b; Bussmann et al., 2017a; Bussmann et al., 2017b; Jakeli et al., 2018; Bussmann et al., 2020a; Bussmann et al., 2020b; Kazanci et al., 2020; Kazanci et al., 2021). It was shown that nineteen taxa had similar uses to those in our study: *Plantago major* subsp. *major* (acne, cough, sore throat, wound, stomach ulcer), *Pinus sylvestris* var. *hamata* (cough, bronchitis, lung diseases, wound)*, Rosa canina* (common cold, cough, urinary system diseases, haemorrhoids)*, Urtica dioica* L. (diabetes, rheumatism, hair loss, haemorrhoids), *Hypericum montbretii* Spach (antifungal, itch, wound)*, Equisetum arvense* L. (diuretic, kidney diseases)*, Hypericum perforatum* (cough, wound)*, Plantago lanceolata* L. (cough, wound), *Viscum album* L. subsp. *album* (diabetes, urinary system diseases), *Abies nordmanniana* subsp. *equi-trojani* (tuberculosis), *Chelidonium majus* (wound)*, Cornus mas* (diarrhoea)*, Crataegus monogyna* Jacq. (cardiovascular system diseases)*, C. orientalis* Pall. ex M.Bieb. subsp. *orientalis* (cardiovascular system diseases)*, C. rhipidophylla* Gand. var. *rhipidophylla* (cardiovascular system diseases)*, Malva neglecta* (haemorrhoids)*, Matricaria chamomilla* (cough), *Rumex crispus* (constipation) and *Tilia rubra* subsp. *caucasica* (sore throat)*.* Among these, *Plantago, Pinus, Rosa* and *Urtica* species take the lead with four and five uses, respectively.

The great majority of species reported in studies are found to have various parts (leaf, root, stem, flower, fruit, seed, etc.) of the plants used for medicinal purposes, while *Crataegus rhipidophylla* var. *rhipidophylla* (fruits), *Rosa canina* (fruits) and *Tilia rubra* subsp. *caucasica* (flowers) are found to have only one part of the plant.

While leaves are the most used plant parts, *Urtica dioica* and *Plantago major* subsp. *major* accounted for the majority of the leaf uses in all studies.

The most common way of using plants medicinally in all studies is preparing infusion/decoction. They are followed by fresh use, mixture and maceration.

Except for the external use of the latex of *Chelidonium majus*, the common method of application in studies in general is internally.

### Review of local plant names

Some of the vernacular names of many plants used medicinally in the area were recorded for the first time throughout northern Anatolia by this study according to “Tuzlaci, (2011)” and the aforementioned literature sources. These were papatya (*Anthemis sintenisii*); geven (*Astracantha microptera*), karaağaç (*Carpinus orientalis* Mill.); çizme otu, terme otu (*Chelidonium majus*); destebozan, yaban karakavuğu (*Cichorium intybus* L.); öküzgötü, yemişen (*Crataegus orientalis* Pall. ex M.Bieb. subsp. *orientalis, C. azarolus* var*. pontica* (K.Koch) K.I.Chr.*, C. tanacetifolia*); sahlep, salep (*Dactylorhiza romana* (Seb.) Soó subsp. *romana*); salep (*D. urvilleana* (Steud.) H. Baumann et Künkele); beygir otu, kındıra, ulasır otu (*Equisetum arvense, E. palustre, E. telmateia*); köpek sütü, sütleven, sütlü ot (*Euphorbia esula* subsp*. tommasiniana* (Bertol.) Kuzmanov, *Euphorbia seguieriana* subsp. *niciciana*); köpek sütü, sütleven (*Euphorbia seguieriana* subsp. *seguieriana*); caba otu (*Hyoscyamus niger* L.); dağ çayı, sarı ot (*Hypericum montbretii*); dağ çayı, kantaron, sarı ot (*Hypericum orientale*); dağ çayı (*Hypericum perforatum*); ömür ardıcı (*Juniperus excelsa* M. Bieb*.*); sütleğen otu (*Lactuca serriola*); bertik otu (*Marrubium anisodon* K. Koch*, M. vulgare* L.); diken otu (*Onopordum acanthium, O. tauricum*); sahlep (*Orchis coriophora, O. morio* subsp. *morio, O. purpurea, O. simia*, *O. tridentata*); siğil yaprağı (*Plantago lanceolata*); siğil yaprağı, sinir yaprağı (*Plantago major* subsp. *intermedia* (Gilib.) Lange); menevşe (*Primula vulgaris* Huds.); ala erik, örük (*Prunus divaricata* subsp. *divaricata* Ledeb.); kel ahlat (*Pyrus elaeagnifolia* subsp. *elaeagnifolia* Pall.); kara meşe (*Quercus infectoria* subsp. *boissieri*); boz meşe (*Quercus petraea* subsp*. iberica*); kışburnu (*Rosa canina*); kır böğürtleni (*Rubus canescens* var. *canescens, R. canescens* var. *glabratus, R. hirtus*, *R. sanctus*); acımık otu (*Rumex crispus*); dağ yaprağı, kabalak (*Salvia sclarea*); acı şabla, şabla otu, şapla, şaplak (*Salvia tomentosa*); şabla, şabla otu, şapla, şaplak (*Salvia verticillata* subsp. *amasiaca*); adaçayı (*Sideritis amasiaca*); dağ kekiği (*Thymus longicaulis* subsp. *longicaulis* C. Presl, *T. praecox* Opiz); hurç (*Viscum album* subsp. *album*); and hurç, purç (*Viscum album*. subsp. a*ustriacum*).

As with the names above, the newly-recorded local names were generally derived from such as the plant’s physical appearance (*sarı ot* = yellow herb; *diken otu* = thorn herb), the plant’s habitat (*dağ kekiği* = mountain thyme; k*ır böğürtleni* = prairie blackberry) or the plant’s usage (*bertik otu* = bruise herb; *terme otu* = eczema herb). It was occasionally observed that the local inhabitants used the same vernacular name to refer to two or more different plant species (Table 5).

It can be said that giving the same name to different species of a genus (gelincik: *Papaver dubium*–*P. rhoeas*; dağ çayı, kantaron, sarı çiçek, sarı ot: *Hypericum montbreti*–*H. orientale*–*H. perforatum*) or to different taxa (sütlü ot: *Euphorbia* spp.–*Lactuca serriola*; papatya: *Anthemis* spp.–*Cota tinctoria* var. *pallida* - *Matricaria chamomilla* - *Tripleurospermum rosellum* var. *album*; etc.) serving the same purpose of use causes this situation. Also, one-third of the whole plants named with a single Turkish word. This could be attributed to the similar cultural backgrounds of the communities living in these villages.

### Quantitative findings

Several studies in northern Anatolia (Polat et al., 2015; Eminagaoğlu et al., 2017; Karci et al., 2017; Yesilyurt et al., 2017; Gurbuz et al., 2019; Karakose et al., 2019; Kazanci et al., 2020; Ergul Bozkurt, 2021; Gurdal and Ozturk, 2021; Akbulut et al., 2022; Karakose, 2022a; Sener et al., 2022) used F_IC_ and/or CI calculations.

Comparing the top three F_IC_ values, the category of respiratory system disorders had the highest rank in both our study (0.95) and three other studies (0.83, 0.76, 0.86) (Karakose et al., 2019; Akbulut et al., 2022; Karakose, 2022a), respectively. In other studies, however, it had a lower rank. The “common cold” is an affliction in this category common to our study and three other studies. Also, *Rosa canina* and *Pinus sylvestris* var. *hamata* are taxa common to our study and two other studies (Karakose et al., 2019; Karakose, 2022a).

While the category of skin and subcutaneous tissue disorders ranked second in our study (0.94), it ranked first in four other studies (0.62, 0.76, 0.75, 0.96) (Polat et al., 2015; Gurbuz et al., 2019; Kazanci et al., 2020; Sener et al., 2022), respectively, and below second in other studies.

While “wound” is an ailment found in both our study and another study (Kazanci et al., 2020), *Plantago* spp. use to treat wounds is found in our study and in three other studies (Gurbuz et al., 2019; Kazanci et al., 2020; Sener et al., 2022).

Finally, the nervous system category held third place in our study (0.92) but held first place (1.00) in another study (Yesilyurt et al., 2017). The plants used in this category differ, but they are used as sedatives in both our study and another study (Yesilyurt et al., 2017).

**FIGURE 9:**
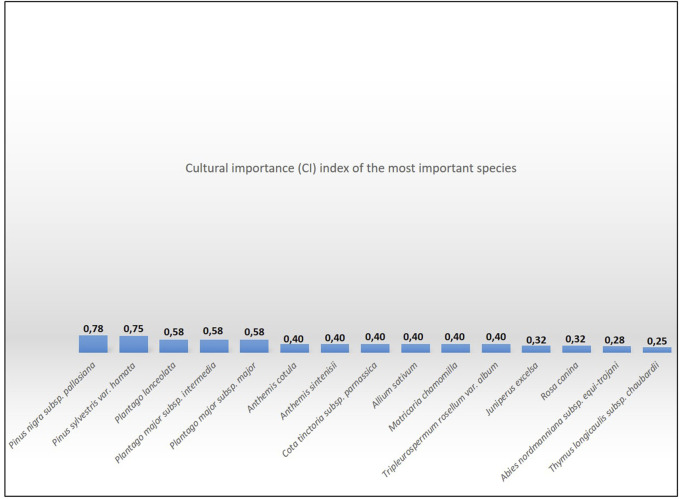
Cultural importance (CI) index of the 15 most important species in Taşköprü.

In a comparison of CI values, *Plantago* spp. were in first place (CI: 1.26) in “Kazanci (2020),” while they were among the first five (CI: 0.58) in our study (Figure 9).

## Conclusion

This pharmaceutical ethnobotanical study was conducted in the whole of Taşköprü District. Besides 20 of the 101 identified plant taxa were recorded as medicinal plants for the first time, a total of 303 new therapeutic uses were documented in this study. This record of the medicinal uses of plants in Taşköprü should be considered evidence that the local inhabitants still derive benefits from nature.

However, it is indisputable that these benefits will gradually decrease. Nine of 101 taxa were recorded as endemic in this study. All of these endemics are conservation priorities and are listed as least concern (LC) except for *Sideritis amasiaca* and *Tripleurospermum rosellum* var. *album*, which are listed vulnerable (VU). Protection measures should be implemented as soon as possible in order to secure the future of these taxa. Medicinal plants are especially important for Turkey. The planting, harvesting, production and trade of these plants should be regulated in order to both control the gathering of wild medicinal plants and prevent damage to biodiversity. In our research region, wild orchids are the first plants that come to mind in this regard. It is also possible for these plants to be cultured in certain regions and to make a greater contribution to the economy.

This study reflects the richness of the region’s flora, shows that the villages throughout the region still benefit from plants, and demonstrates that plants are still considered a viable alternative to modern methods of treatment.

Furthermore, this study showed that less of the traditional knowledge of medicinal plants was transmitted from the second generation to the third generation than was transmitted from the first generation to the second generation. Consequently, this traditional knowledge will inevitably decrease and ultimately disappear if further studies are not urgently undertaken and completed. Simply put, the elderly members of these communities can no longer recall all of their knowledge of medicinal plants, and they are dying before they can pass on what they do recall to the younger members.

In order to prevent this situation and reach young people, booklets on the subject could be published, this issue could be mentioned at local meetings or festivals, or some activities could be carried out using social media, where this audience is of great interest.

In addition, with these ethnobotanical studies, traditional knowledge will be preserved and a basic resource will be provided for further specialized studies of this subject for the discovery of drugs’ active ingredients or new drugs.

## Data Availability

The raw data supporting the conclusion of this article will be made available by the authors, without undue reservation.
