# Supplementary material for: An Ethnobotanical study of medicinal plants in Taşköprü (Kastamonu–Turkey)
Source: Front Pharmacol. 2022 Oct 20;13:984065. doi: 10.3389/fphar.2022.984065 (PMC9630845; doi:10.3389/fphar.2022.984065)
Supplement: Supplementary file 1 [file Table1.docx]

| **Supplementary Table S1. Folk medicinal plants of Taşköprü (Kastamonu/TURKEY)** | | | | | | | | | |
| --- | --- | --- | --- | --- | --- | --- | --- | --- | --- |
| **Botanical name, Family and Specimen number**  (new plant records in bold) | **Local name** (in Turkish) | **Plant part used** | **Ailments treated/ Therapeutic effect**  (new uses in bold) | **Preparation** | **Administration, dosage** | **Rpt.** | **CI** | **References from North Anatolia** | **References from Balkans and Caucasus** |
| **AMARANTHACEAE** | | | | | | | | | |
| *Beta vulgaris* L. ^a^, (Obs.) [Syn.: *B. vulgaris* var. *altissima* Döll] | Pancar, şeker pancarı | Roots | Cough | Molasses (+lemon and olive oil) | Int. | 3 | 0.04 | Cough (44)  Sinusitis (38) (17,20,26,31,39,40,44)^b^ | − |
|  |  |  | **Bronchitis** | Molasses (mixed with melted butter) | Int. | 2 |  |  |  |
|  |  |  | Sinusitis | The bottom is cut and filled with olive oil after carved | The liquid dripping from the bottom is dropped into the nostrils (1 drop for each) | 2 |  |  |  |
| **AMARYLLIDACEAE** | | | | | | | | | |
| *Allium cepa* L.^a^, (Obs.) | Soğan | Bulbs | Boil | Roasted | Ext. | 2 | 0.03 | Boil (2,4,21,27,31,44,50)  Earache (31)  (3,4,7,9,10,17,20,21,23,26,27,31,38,39,40,43,44,50)^b^ | (33)^b^ |
|  |  |  | Earache | Heated | Inserted in the ear | 4 |  |  |  |
| *Allium sativum* L.^a^ MARE 19041 | Sarımsak | Bulbils | Sore throat | − | Int. | 4 | 0.40 | Hair loss (9,14,20,26,31,43)  Toothache (20,31)  Hypertension (7,9,10,20,21,26,27,31,39,40,44,49)  Haemorrhoids (20,31)  Earache (21,27,39,40,44)  Sore throat (39)  (2,7,9,10,20,21,26,27,31,39,40,41,43,44,49)^b^ | (22,52)^b^ |
|  |  |  | Hypertension | − | Int. | 5 |  |  |  |
|  |  |  | **Fever** | − | Int. | 5 |  |  |  |
|  |  |  | **Heartburn** | Crushed and boiled with milk | Int. | 3 |  |  |  |
|  |  |  | Earache | Heated | Inserted in the ear | 16 |  |  |  |
|  |  |  | Hair loss | Crushed and mixed with olive oil | Ext. | 27 |  |  |  |
|  |  |  | Toothache | Crushed | Ext. | 7 |  |  |  |
|  |  |  | Haemorrhoids | Crushed | Ext. | 2 |  |  |  |
|  |  |  | **Animal (dog, snake) bite** | Crushed | Ext. | 6 |  |  |  |
|  |  |  | **Mouth sore** | Chewed | Ext. | 4 |  |  |  |
| **APIACEAE** | | | | | | | | | |
| *Petroselinum crispum* (Mill.) Fuss ^s^, MARE 18428 [Syn.: *P. crispum* (Miller) A.W Hill] | Maydanoz | Aerial parts | Kidney diseases | Decoction | Int. | 1 | 0.01 | Kidney diseases (40) (3,4,6,7,9,17,20,21,27,29,30, 38,39,40,43,44)^b^ | (19)^b^ |
| **ASTERACEAE** | | | | | | | | | |
| *Anthemis cotula* L., MARE 18260, 19002, 19031 | Papatya | Capitula | Abdominal pain | Decoction | Int. | 4 | 0.40 | Abdominal pain (2,15)  Common cold (45)  Shortness of breath (45) (2,18,31,43,45)^b^ | − |
|  |  |  | **Nasal congestion** | Decoction | Inhalation | 2 |  |  |  |
|  |  |  | Shortness of breath | Decoction | Inhalation | 4 |  |  |  |
|  |  |  | Common cold | Infusion | Int. | 21 |  |  |  |
|  |  |  | **Sedative** | Infusion | Int. | 12 |  |  |  |
|  |  |  | **Constipation** | Infusion | Int. | 3 |  |  |  |
|  |  |  | **Cough** | Infusion | Int. | 10 |  |  |  |
|  |  |  | **Shortness of breath** | Infusion | Int. | 18 |  |  |  |
|  |  | Aerial parts | **Haemorrhoids** | Decoction | Int. | 3 |  |  |  |
|  |  |  | **Urinary system diseases** | Infusion | Int. | 2 |  |  |  |
| ***Anthemis sintenisii*** Freyn, MARE 18153 | Papatya | Capitula | **Abdominal pain** | Decoction | Int. | 4 | 0.40 | − | − |
|  |  |  | **Nasal congestion** | Decoction | Inhalation | 2 |  |  |  |
|  |  |  | **Shortness of breath** | Decoction | Inhalation | 4 |  |  |  |
|  |  |  | **Common cold** | Infusion | Int. | 21 |  |  |  |
|  |  |  | **Sedative** | Infusion | Int. | 12 |  |  |  |
|  |  |  | **Constipation** | Infusion | Int. | 3 |  |  |  |
|  |  |  | **Cough** | Infusion | Int. | 10 |  |  |  |
|  |  |  | **Shortness of breath** | Infusion | Int. | 18 |  |  |  |
|  |  | Aerial parts | **Haemorrhoids** | Decoction | Int. | 3 |  |  |  |
|  |  |  | **Urinary system diseases** | Infusion | Int. | 2 |  |  |  |
| *Cota tinctoria* var. *pallida* (DC.) Özbek et Vural, MARE 18209, 18312, 18381 [Syn.: *Anthemis tinctoria* var. *pallida* DC.] | Papatya | Capitula | **Abdominal pain** | Decoction | Int. | 4 | 0.40 | Shortness of breath (18)  (18)^b^ | − |
|  |  |  | **Nasal congestion** | Decoction | Inhalation | 2 |  |  |  |
|  |  |  | **Shortness of breath** | Decoction | Inhalation | 4 |  |  |  |
|  |  |  | **Common cold** | Infusion | Int. | 21 |  |  |  |
|  |  |  | **Sedative** | Infusion | Int. | 12 |  |  |  |
|  |  |  | **Constipation** | Infusion | Int. | 3 |  |  |  |
|  |  |  | **Cough** | Infusion | Int. | 10 |  |  |  |
|  |  |  | Shortness of breath | Infusion | Int. | 18 |  |  |  |
|  |  | Aerial parts | **Haemorrhoids** | Decoction | Int. | 3 |  |  |  |
|  |  |  | **Urinary system diseases** | Infusion | Int. | 2 |  |  |  |
| *Cichorium intybus* L., MARE 18245, 18383, 18976 | Karakavuk, yaban karakavuğu, destebozan | Aerial parts | **Constipation** | − | Int. | 4 | 0.03 | Diabetes (16)  (4,9,11,14,16,20,31,44,46,49,51)^b^ | (19,22,24,33,47,52,54)^b^ |
| *Helianthus* *tuberosus* L.^a^, MARE 18334 | Yer elması | Tubers | **Liver diseases** | − | Int. | 2 | 0.01 | (11,20,39,40,44)^b^ | (33,55)^b^ |
| *Helichrysum* *aucheri* Boiss., MARE 18395 [Syn.: *H.* *arenarium* subsp. *aucheri* (Boiss.) Davis et Kupicha] | Koyungözü, sarılık otu | Aerial parts | Cough | Decoction | Int. | 2 | 0.02 | Cough (40)  (21,40)^b^ | − |
|  |  | Capitula | **Icterus (in children)** | Decoction | Int. | 2 |  |  |  |
| ***Lactuca serriola*** L., MARE 18285, 19025 | Sütlü ot, sütleğen otu | Latex | **Wart** | − | Ext. (2-3 drops-once a day) | 1 | 0.01 | − | − |
| *Matricaria chamomilla* Blanco, MARE 18130, 19001 [Syn.: *M. chamomilla* var. *recutita* (L.) Grierson] | Papatya | Capitula | Abdominal pain | Decoction | Int. | 4 | 0.40 | Common cold (10,11,21,25,44)  Cough (6,10,15,16,44)  Sedative (28,44)  Abdominal pain(41)  Constipation (44) Urinary sys. dis. (44)  Haemorrhoids (14,44) (6,10,11,14,15,16,21,28,44)^b^ | Cough (52)  (22,24,33,52,53,54)^b^ |
|  |  |  | **Nasal congestion** | Decoction | Inhalation | 2 |  |  |  |
|  |  |  | **Shortness of breath** | Decoction | Inhalation | 4 |  |  |  |
|  |  |  | Common cold | Infusion | Int. | 21 |  |  |  |
|  |  |  | Sedative | Infusion | Int. | 12 |  |  |  |
|  |  |  | Constipation | Infusion | Int. | 3 |  |  |  |
|  |  |  | Cough | Infusion | Int. | 10 |  |  |  |
|  |  |  | **Shortness of breath** | Infusion | Int. | 18 |  |  |  |
|  |  | Aerial parts | Haemorrhoids | Decoction | Int. | 3 |  |  |  |
|  |  |  | Urinary system disease**s** | Infusion | Int. | 2 |  |  |  |
| ***Onopordum* *acanthium*** L., MARE 18139, 18435 | Diken otu | Capitula | **Heart diseases** | Decoction | Int. | 3 | 0.03 | − | − |
|  |  | Fruits | **Malaria** | Decoction | Int. | 2 |  |  |  |
| *Onopordum* *tauricum* Willd., MARE 18263 | Diken otu | Capitula | **Heart diseases** | Decoction | Int. | 3 | 0.03 | (14)^b^ | − |
|  |  | Fruits | **Malaria** | Decoction | Int. | 2 |  |  |  |
| *Petasites hybridus* (L.) G. Gaertn., B. Mey. et Scherb., MARE 18217 | Kabalak | Leaves | Wound | − | Ext. (wrapped in a cloth) | 2 | 0.01 | Wound (10)  (8,10,13,16,21,40)^b^ | − |
| ***Tripleurospermum* *rosellum*** (Boiss. et Orph.) Hayek var. ***album*** E. Hossain, MARE 18086, 18106, 18877, 18885, 18890 | Papatya | Capitula | **Abdominal pain** | Decoction | Int. | 4 | 0.40 | − | − |
|  |  |  | **Nasal congestion** | Decoction | Inhalation | 2 |  |  |  |
|  |  |  | **Shortness of breath** | Decoction | Inhalation | 4 |  |  |  |
|  |  |  | **Common cold** | Infusion | Int. | 21 |  |  |  |
|  |  |  | **Sedative** | Infusion | Int. | 12 |  |  |  |
|  |  |  | **Constipation** | Infusion | Int. | 3 |  |  |  |
|  |  |  | **Cough** | Infusion | Int. | 10 |  |  |  |
|  |  |  | **Shortness of breath** | Infusion | Int. | 18 |  |  |  |
|  |  | Aerial parts | **Haemorrhoids** | Decoction | Int. | 3 |  |  |  |
|  |  |  | **Urinary system diseases** | Infusion | Int. | 2 |  |  |  |
| **BETULACEAE** | | | | | | | | | |
| *Carpinus orientalis* Mill., MARE 18166, 18372, 18952 | Karaağaç | Roots | **Stomach ailments** | Decoction (cut into little pieces) | Int. | 2 | 0.01 | (3)^b^ | − |
| **CORNACEAE** | | | | | | | | | |
| *Cornus mas* L., MARE 18167, 18427, 18985 | Kiren | Fruits | Diabetes | − | Int. | 17 | 0.28 | Diarrhoea (2,4,8,10,11,13,20,27,31,34,40,44)  Diabetes (4,8,21,26,31,40,44)  (2,4,8,9,10,11,14,17,20,27,31,39,40)^b^ | Diarrhoea (34)  (34)^b^ |
|  |  |  | Diabetes | Marmalade | Int. | 36 |  |  |  |
|  |  |  | Diarrhoea | − | Int. (a handful-twice a day) | 2 |  |  |  |
| **CUCURBITACEAE** | | | | | | | | | |
| *Citrullus lanatus* (Thunb.) Matsum. et Nakai^a^, (Obs.) | Karpuz | Fruit juice | **Earache** | − | Dropped (3-4 drops) into the ear | 1 | 0.01 | (26,31,40)^b^ | − |
| *Ecballium* *elaterium* (L.) A. Rich. Cucurbitaceae, MARE 20412 | Yabani kavun | Fruit juice | Haemorrhoids | − | Ext. | 2 | 0.01 | Haemorrhoids (6,8,10,11,14,26, 38)  (2,6,7,8,9,10,11,12,14,15,16,26,29,31,38)^b^ | − |
| **CUPRESSACEAE** | | | | | | | | | |
| *Juniperus excelsa* M. Bieb., MARE 18180, 18328 | Ardıç, ömür ardıcı | Cones | **Shortness of breath** | Decoction | Int. | 6 | 0.32 | Cough (12)  Haemorrhoids (37)  (2,37)^b^ | − |
|  |  |  | Cough | Decoction | Int. | 6 |  |  |  |
|  |  |  | **Stomachache** | Decoction | Int. | 4 |  |  |  |
|  |  |  | **Eczema** | Decoction | Int. | 2 |  |  |  |
|  |  | Roots | **Diabetes** | Decoction | Int. | 4 |  |  |  |
|  |  | Resin (obtained from roots) | **Cough** | − | Swallowed (2-3 pieces a day) | 4 |  |  |  |
|  |  |  | **Expectorant** | − | Swallowed (2-3 pieces a day) | 4 |  |  |  |
|  |  | Tar | **Toothache** | − | Dropped onto the teeth | 2 |  |  |  |
|  |  |  | **Rheumatism** | − | Ext. | 6 |  |  |  |
|  |  |  | Haemorrhoids | − | Ext. | 3 |  |  |  |
|  |  |  | **Low back complaint** | − | Ext. | 1 |  |  |  |
|  |  |  | **Wound** | − | Ext. | 5 |  |  |  |
|  |  |  | **Scabies** | − | Ext. | 17 |  |  |  |
| *Juniperus oxycedrus* L., MARE 18143, 18205, 18283, 18319, 18860, 18895, 19032 [Syn.: *J. oxycedrus* L. subsp. *oxycedrus*] | Ardıç | Cones | Cough | Decoction | Int. | 6 | 0.23 | Haemorrhoids (1,2,4,8,29)  Stomachache (8)  Cough (2,4)  Eczema (4,10,20)  Rheumatism (20,28,44)  Diabetes (11, 38,44)  Wound (28)  (1,2,4,6,10,11,20,28,29,42,44)^b^ | − |
|  |  |  | Stomachache | Decoction | Int. | 4 |  |  |  |
|  |  |  | Eczema | Decoction | Int. | 2 |  |  |  |
|  |  | Roots | Diabetes | Decoction | Int. | 4 |  |  |  |
|  |  | Resin (obtained from roots) | Cough | − | Swallowed (2-3 pieces a day) | 4 |  |  |  |
|  |  |  | **Expectorant** | − | Swallowed (2-3 pieces a day) | 4 |  |  |  |
|  |  | Tar | **Toothache** | − | Dropped onto the teeth | 2 |  |  |  |
|  |  |  | Rheumatism | − | Ext. | 6 |  |  |  |
|  |  |  | Haemorrhoids | − | Ext. | 3 |  |  |  |
|  |  |  | **Low back complaint** | − | Ext. | 1 |  |  |  |
|  |  |  | Wound | − | Ext. | 5 |  |  |  |
|  |  |  | Eczema | − | Ext. | 5 |  |  |  |
| **EQUISETACEAE** | | | | | | | | | |
| *Equisetum* *arvense* L., MARE 18448, 19074 | Kındıra, ulasır otu, kırkkilit otu, beygir otu | Aerial parts | Diuretic | Decoction | Int. | 2 | 0.02 | Diuretic (16)  Kidney dis. (16,44)  (16,17,18,20,34,43,44,47)^b^ | Diuretic (5)  Kidney dis. (24,34,54)  (5,34,38,52,53,55)^b^ |
|  |  |  | Kidney diseases | Decoction | Int. | 2 |  |  |  |
| ***Equisetum* *palustre*** L., MARE 18406 | Kındıra, ulasır otu, kırkkilit otu, beygir otu | Aerial parts | **Diuretic** | Decoction | Int. | 2 | 0.02 | − | − |
|  |  |  | **Kidney diseases** | Decoction | Int. | 2 |  |  |  |
| *Equisetum* *telmateia* Ehrh. Equisetaceae, MARE 18329 | Kındıra, ulasır otu, kırkkilit otu, beygir otu | Aerial parts | Diuretic | Decoction | Int. | 2 | 0.02 | Diuretic (6,7,21,40)  Kidney dis. (10,31)  (3,6,7,8,9,10,15,21,27,31,40)^b^ | (55)^b^ |
|  |  |  | Kidney diseases | Decoction | Int. | 2 |  |  |  |
| **EUPHORBIACEAE** | | | | | | | | | |
| *Euphorbia esula* subsp. *tommasiniana* (Bertol.) Kuzmanov, MARE 18315, 18937 [Syn.: *E. virgata* Waldst. et Kit.] | Sütleğen, köpek sütü, sütleven, sütlü ot | Latex | **Wound** | − | Ext. (1-2 drops) | 5 | 0.15 | (37)^b^ | − |
|  |  |  | **Wart** | − | Ext. (1-2 drops) | 18 |  |  |  |
|  |  |  | **Psoriasis** | − | Ext. (1-2 drops) | 2 |  |  |  |
|  |  |  | **Boil** | − | Ext. (1-2 drops) | 3 |  |  |  |
|  |  | Aerial parts | **Cut** | Cut into little pieces and boiled with milk | Ext. (as poultice, wrapped in a cloth) | 2 |  |  |  |
| ***Euphorbia* *seguieriana*** subsp. ***niciciana*** (Borbás ex Novák) Rech. f., MARE 18379, 18970 | Sütleğen, köpek sütü, sütleven, sütlü ot | Latex | **Wound** | − | Ext. (1-2 drops) | 5 | 0.15 | − | − |
|  |  |  | **Wart** | − | Ext. (1-2 drops) | 18 |  |  |  |
|  |  |  | **Psoriasis** | − | Ext. (1-2 drops) | 2 |  |  |  |
|  |  |  | **Boil** | − | Ext. (1-2 drops) | 3 |  |  |  |
|  |  | Aerial parts | **Cut** | Cut into little pieces and boiled with milk | Ext. (as poultice, wrapped in a cloth) | 2 |  |  |  |
| *Euphorbia* *seguieriana* Neck., MARE 18100, 18256, 18316 [Syn.: *E.* *seguieriana* Neck. subsp. *seguieriana*] | Sütleğen, köpek sütü, sütleven, sütlü ot | Latex | **Wound** | − | Ext. (1-2 drops) | 5 | 0.15 | (4,20)^b^ | − |
|  |  |  | **Wart** | − | Ext. (1-2 drops) | 18 |  |  |  |
|  |  |  | **Psoriasis** | − | Ext. (1-2 drops) | 2 |  |  |  |
|  |  |  | **Boil** | − | Ext. (1-2 drops) | 3 |  |  |  |
|  |  | Aerial parts | **Cut** | Cut into little pieces and boiled with milk | Ext. (as poultice, wrapped in a cloth) | 2 |  |  |  |
| **FABACEAE** | | | | | | | | | |
| *Astracantha* *microcephala* (Willd.) Podlech, MARE 18165, 18267, 18968, 19053, 19087 [Syn.: *Astragalus microcephalus* Willd.] | Geven | Aerial parts | **Scabies** | Decoction | Ext. | 2 | 0.01 | (32,34,47)^b^ | (34)^b^ |
| **FAGACEAE** | | | | | | | | | |
| ***Quercus infectoria*** subsp. ***veneris*** (A.Kern.) Meikle, MARE 18096, 18179, 18291, 18313, 18893, 18904 [Syn.: *Q.* *infectoria* subsp. *boissieri* (Reut.) O. Schwarz] | Kara meşe, meşe | Fruits | **Wound** | Roasted | Ext. | 3 | 0.03 | − | − |
|  |  | Sap (obtained from young branches through exposing to fire) | **Earache** | − | Dropped (3-4 drops) into the ear | 2 |  |  |  |
| ***Quercus* *macranthera*** subsp. ***syspirensis*** (K. Koch) Menitsky, MARE 18156 | Meşe | Fruits | **Wound** | Roasted | Ext. | 3 | 0.03 | − | − |
|  |  | Sap (obtained from young branches through exposing to fire) | **Earache** | − | Dropped (3-4 drops) into the ear | 2 |  |  |  |
| *Quercus petraea* subsp*. iberica* (Steven ex M.Bieb.) Krassiln*.*, MARE 18182, 19003, 19086 | Boz meşe, meşe | Fruits | Wound | Roasted | Ext. | 3 | 0.03 | Wound (38,40)  (23,38,40)^b^ | (34,47)^b^ |
|  |  | Sap (obtained from young branches through exposing to fire) | **Earache** | − | Dropped (3-4 drops) into the ear | 2 |  |  |  |
| **HYPERICACEAE** | | | | | | | | | |
| *Hypericum montbretii* Spach, MARE 18330, 18374 | Dağ çayı, kantaron, sarı çiçek, sarı ot | Flowering parts | Antifungal | Decoction | Int. | 1 | 0.12 | Antifungal (34)  Itch (34)  Wound (34)  (15,16,31,34)^b^ | Antifungal (34)  Wound (34)  Itch (34)  (34,54)^b^ |
|  |  |  | **Common cold** | Infusion | Int. | 2 |  |  |  |
|  |  |  | Itch | Decoction | Ext. | 1 |  |  |  |
|  |  |  | **Prostate ailments** | Decoction | Int. | 2 |  |  |  |
|  |  |  | **Cough** | Decoction | Int. | 2 |  |  |  |
|  |  |  | **Analgesic** | Oleate | Ext. | 3 |  |  |  |
|  |  |  | **Rheumatic pain** | Oleate | Ext. | 3 |  |  |  |
|  |  | Aerial parts | Wound | Oleate | Ext. | 2 |  |  |  |
|  |  |  | **Burn** | Oleate | Ext. | 2 |  |  |  |
|  |  |  | **Back complaint** | Oleate | Ext. | 2 |  |  |  |
|  |  |  | **Rheumatic pain** | Oleate | Ext. | 2 |  |  |  |
| *Hypericum orientale* L., MARE 18360 | Dağ çayı, kantaron, sarı çiçek, sarı ot | Flowering parts | **Antifungal** | Decoction | Int. | 1 | 0.12 | (9,34,46)^b^ | − |
|  |  |  | **Common cold** | Infusion | Int. | 2 |  |  |  |
|  |  |  | **Itch** | Decoction | Ext. | 1 |  |  |  |
|  |  |  | **Prostate ailments** | Decoction | Int. | 2 |  |  |  |
|  |  |  | **Cough** | Decoction | Int. | 2 |  |  |  |
|  |  |  | **Analgesic** | Oleate | Ext. | 3 |  |  |  |
|  |  |  | **Rheumatic pain** | Oleate | Ext. | 3 |  |  |  |
|  |  | Aerial parts | **Wound** | Oleate | Ext. | 2 |  |  |  |
|  |  |  | **Burn** | Oleate | Ext. | 2 |  |  |  |
|  |  |  | **Back complaint** | Oleate | Ext. | 2 |  |  |  |
|  |  |  | **Rheumatic pain** | Oleate | Ext. | 2 |  |  |  |
| *Hypericum perforatum* L., MARE 18251, 18308, 18369, 18378, 18415, 18963 | Dağ çayı, kantaron, sarı çiçek, sarı ot | Flowering parts | Antifungal | Decoction | Int. | 1 | 0.18 | Wound (1,6,8,10,11,12,14,16,18,21,34,38,40,41,43,44)  Cough (34,45)  Burn (6,10,18,21,41)  Analgesic (38,40,44)  Rheumatism (8,27)  Common cold (6,10,26,28,38)  Antifungal (10)  Prostate ailments (11)  (1,6,8,9,10,11,12,14,15,16,21,25,26,27,28,29,30,32,34,37,38,40,43,44,45,47,52)^b^ | Wound (34)  Cough (34)  (22,24,33,34,53,54)^b^ |
|  |  |  | Common cold | Infusion | Int. | 2 |  |  |  |
|  |  |  | **Itch** | Decoction | Ext. | 1 |  |  |  |
|  |  |  | Prostate ailments | Decoction | Int. | 2 |  |  |  |
|  |  |  | Cough | Decoction | Int. | 2 |  |  |  |
|  |  |  | Analgesic | Oleate | Ext. | 3 |  |  |  |
|  |  |  | Rheumatic pain | Oleate | Ext. | 3 |  |  |  |
|  |  | Aerial parts | Wound | Oleate | Ext. | 2 |  |  |  |
|  |  |  | Burn | Oleate | Ext. | 2 |  |  |  |
|  |  |  | **Back complaint** | Oleate | Ext. | 2 |  |  |  |
|  |  |  | **Rheumatic pain** | Oleate | Ext. | 2 |  |  |  |
| **IRIDACEAE** | | | | | | | | | |
| *Crocus* *ancyrensis* (Herb.) Maw, MARE 18839, 18845 | Sarı çiğdem | Flowers | **Earache** | Decoction | Dropped (2-3 drops) into the ear | 7 | 0.06 | (29)^b^ | − |
|  |  | Sap | **Earache** | Obtained by crushing perigon | Dropped (2-3 drops) into the ear | 4 |  |  |  |
| **JUGLANDACEAE** | | | | | | | | | |
| *Juglans regia* L.^a^, MARE 18162, 18322, 19037 | Ceviz | Leaves | Tonsillitis | − | Ext. (wrapped in a cloth) | 3 | 0.04 | Tonsillitis (23)  Hair strengthener (23)  (2,3,4,6,7,9,10,11,12,13,14,15,17,18,20,21,25,27,32,34,38,39,40,41,43,44,46,50)^b^ | (19,34,54)^b^ |
|  |  | Immature fruits | **Purgative** | − | Int. | 2 |  |  |  |
|  |  | Pericarp | Hair strengthener | Decoction | Ext. (as rinse water) | 2 |  |  |  |
| **LAMIACEAE** | | | | | | | | | |
| ***Marrubium anisodon*** K. Koch, MARE 18351 | Bertik otu | Leaves | **Sprain** | − | Ext. (wrapped in a cloth) | 3 | 0.02 | − | − |
| ***Marrubium vulgare*** L., MARE 19051 | Bertik otu | Aerial parts | **Sprain** | Crushed | Ext. | 2 | 0.01 | − | − |
| *Mentha* x *piperita* L.^a^, MARE 18422 | Nane | Aerial parts | Common cold | Decoction (+lemon juice) | Int. (a glass-twice a day) | 16 | 0.08 | Common cold (29,41)  (27,29,41,44)^b^ | (22,52,53)^b^ |
| *Origanum vulgare* L., MARE 18340 | Dağ çayı | Aerial parts | **Immunostimulant** | Infusion | Int. (a glass-once a day) | 2 | 0.01 | (8,14,18,21,25,27,30, 34,38,45,47)^b^ | (19,24,34,47,53,54)^b^ |
| *Salvia* *sclarea* L., MARE 18254, 18306, 18426, 18986 | Dağ yaprağı, kabalak | Leaves | **Bruise** | − | Ext. (wrapped in a cloth) | 2 | 0.14 | Common cold (25)  (20,25)^b^ | − |
|  |  |  | **Cut** | Crushed | Ext. (wrapped in a cloth) | 3 |  |  |  |
|  |  |  | **Haemostatic** | Crushed | Ext. (wrapped in a cloth) | 8 |  |  |  |
|  |  | Aerial parts | Common cold | Infusion | Int. | 8 |  |  |  |
|  |  |  | **Stomach ulcer** | Infusion | Int. | 3 |  |  |  |
|  |  |  | **Digestive** | Infusion | Int. (a glass-after meals) | 3 |  |  |  |
| *Salvia* *tomentosa* Mill., MARE 18188, 18324, 18418, 19059 | Acı şabla, adaçayı, şabla, şabla otu, şapla, şaplak | Leaves | **Bruise** | − | Ext. (wrapped in a cloth) | 2 | 0.14 | Common cold (40)  Haemostatic (39)  Digestive (39)  (32,39,40)^b^ | − |
|  |  |  | **Cut** | Crushed | Ext. (wrapped in a cloth) | 3 |  |  |  |
|  |  |  | Haemostatic | Crushed | Ext. (wrapped in a cloth) | 8 |  |  |  |
|  |  | Aerial parts | Common cold | Infusion | Int. | 8 |  |  |  |
|  |  |  | **Stomach ulcer** | Infusion | Int. | 3 |  |  |  |
|  |  |  | Digestive | Infusion | Int. (a glass-after meals) | 3 |  |  |  |
| *Salvia* *verticillata* subsp. *amasiaca* (Freyn et Bornm.) Bornm., MARE 18390, 19004, 19021 | Adaçayı, şabla, şabla otu, şapla, şaplak | Leaves | **Bruise** | − | Ext. (wrapped in a cloth) | 2 | 0.07 | (10,20)^b^ | − |
|  |  |  | **Cut** | Crushed | Ext. (wrapped in a cloth) | 3 |  |  |  |
|  |  |  | **Haemostatic** | Crushed | Ext. (wrapped in a cloth) | 8 |  |  |  |
| ***Sideritis* *amasiaca*** Bornm., MARE 18145, 18231, 18279, 18429 | Adaçayı | Aerial parts | **Abdominal pain** | Decoction | Int. | 4 | 0.02 | − | − |
| *Teucrium* *polium* L., MARE 18259, 18289, 18325, 18384, 18959 | Kekik | Aerial parts | **Cough** | Decoction | Int. | 4 | 0.02 | (1,2,4,10,14,15,20,27,34,46)^b^ | − |
| *Thymus longicaulis* subsp. *longicaulis* C. Presl, MARE 18391, 19081, 19082 [Syn.: *T. longicaulis* subsp. *longicaulis* var. *subisophyllus* (Borbás) Jalas] | Dağ kekiği, kekik | Aerial parts | **Sore throat** | Decoction | Int. | 3 | 0.25 | Cough (6,10,11)  Common cold (10)  Abdominal pain (10,14)  Diabetes (11,16)  (6,10,11,15,16)^b^ | − |
|  |  |  | Diabetes | Decoction | Int. | 3 |  |  |  |
|  |  |  | **Weight loss** | Decoction | Int. | 3 |  |  |  |
|  |  |  | **Stomachache** | Decoction | Int. | 4 |  |  |  |
|  |  |  | Cough | Decoction | Int. | 3 |  |  |  |
|  |  |  | **Shortness of breath** | Decoction | Int. | 5 |  |  |  |
|  |  |  | **Expectorant** | Decoction | Int. | 5 |  |  |  |
|  |  |  | **Anthelmintic** | Decoction | Int. | 2 |  |  |  |
|  |  |  | Abdominal pain | Decoction | Int. | 4 |  |  |  |
|  |  |  | **Rheumatism** | Decoction | Int. | 2 |  |  |  |
|  |  |  | Common cold | Infusion | Int. | 13 |  |  |  |
|  |  |  | **Digestive** | Infusion | Int. (a glass-after meals) | 3 |  |  |  |
| *Thymus praecox* Opiz, MARE 18093, 18094, 18160, 18198, 18258, 18311, 18892, 19024 [Syn.: *T. praecox* subsp. *skorpilii* (Velen.) Jalas var. *skorpilii*] | Dağ kekiği, kekik | Aerial parts | **Sore throat** | Decoction | Int. | 3 | 0.23 | Common cold (38)  Cough (38)  Diabetes (9, 38)  Stomachache (9)  (9,38,46)^b^ | − |
|  |  |  | Diabetes | Decoction | Int. | 3 |  |  |  |
|  |  |  | **Weight loss** | Decoction | Int. | 3 |  |  |  |
|  |  |  | Stomachache | Decoction | Int. | 4 |  |  |  |
|  |  |  | Cough | Decoction | Int. | 3 |  |  |  |
|  |  |  | **Anthelmintic** | Decoction | Int. | 2 |  |  |  |
|  |  |  | **Abdominal pain** | Decoction | Int. | 4 |  |  |  |
|  |  |  | **Rheumatism** | Decoction | Int. | 2 |  |  |  |
|  |  |  | **Digestive** | Infusion | Int. (a glass-after meals) | 3 |  |  |  |
|  |  |  | Common cold | Infusion | Int. | 18 |  |  |  |
| **MALVACEAE** | | | | | | | | | |
| *Malva neglecta* Wallr., MARE 18332, 18421, 18440 | Ebegümeci, ebegömeci, ebemgümeci | Aerial parts | Urinary tract infection | Decoction | Int. | 8 | 0.17 | Cough (12,20,38)  Stomachache (2,9,20,44)  Haemorrhoids (29,34,38)  Gyn. dis. (20)  Inflamed wound (20)  Urinary tract inf.(38,45)  Abortive (34,44)  (9,12,20,21,27,37,34,38,44,49,51)^b^ | (33)^b^ |
|  |  |  | Gynecological diseases | Boiled | Ext. (Sit on the poultice) | 6 |  |  |  |
|  |  |  | Haemorrhoids | Decoction | Ext. (Exposed to the vapour) | 3 |  |  |  |
|  |  | Flowers | Cough | Decoction | Int. (a glass-twice a day) | 5 |  |  |  |
|  |  | Leaves | **Stye** | Boiled | Ext. (as poultice) | 2 |  |  |  |
|  |  |  | Inflamed wound | − | Ext. | 3 |  |  |  |
|  |  |  | Stomachache | Waited in the water overnight | Drunk | 3 |  |  |  |
|  |  | Roots | Abortive | − | Inserted in the vagina | 3 |  |  |  |
| *Malva sylvestris* L., MARE 18301 | Ebegümeci, ebegömeci, ebemgümeci | Aerial parts | **Urinary tract infection** | Decoction | Int. | 8 | 0.17 | Cough (8,10,11,12,14,29,40)  Haemorrhoids (10,14,15,23)  Gyn. dis. (10)  Stomachache (10,40)  Abortive (10,44)  (1,3,6,8,10,11,12,13,14,15,16,23,25,28,30,39,40,41,43,44)^b^ | (54)^b^ |
|  |  |  | Gynecological diseases | Boiled | Ext. (Sit on the poultice) | 6 |  |  |  |
|  |  |  | Haemorrhoids | Decoction | Ext. (Exposed to the vapour) | 3 |  |  |  |
|  |  | Flowers | Cough | Decoction | Int. (a glass-twice a day) | 5 |  |  |  |
|  |  | Leaves | **Stye** | Boiled | Ext. (as poultice) | 2 |  |  |  |
|  |  |  | **Inflamed wound** | − | Ext. | 3 |  |  |  |
|  |  |  | Stomachache | Waited in the water overnight | Drunk | 3 |  |  |  |
|  |  | Roots | Abortive | − | Inserted in the vagina | 3 |  |  |  |
| *Tilia rubra* subsp. *caucasica* (Rupr.) V. Engl., MARE 19042 | Ihlamur | Flowers | Common cold | Decoction | Int. (a glass-3 times a day) | 4 | 0.06 | Cough (2,4,18,27, 38,39,40)  Common cold (2,3,4,12,25,27, 32, 38,39,40)  Sore throat (34,39)  (3,18,25,27,32,34,39,40)^b^ | Sore throat (34)  (33,34,54)^b^ |
|  |  |  | Cough | Decoction | Int. (a glass-3 times a day) | 4 |  |  |  |
|  |  |  | Sore throat | Decoction | Int. (a glass-3 times a day) | 4 |  |  |  |
| **ORCHIDACEAE** | | | | | | | | | |
| ***Anacamptis pyramidalis*** (L.) Rich., MARE 18354, 18416 | Sahlep, salep | Tubers | **Constipation** | Decoction | Int. (a glass-once a day) | 3 | 0.04 | − | − |
|  |  |  | **Cough** | Boiled and powdered, then cooked with milk | Int. | 4 |  |  |  |
| ***Dactylorhiza romana*** (Seb.) Soó subsp. ***romana***, MARE 18087, 18088, 18875, 18905, 18921, 18924, 18925, 18926 | Sahlep, salep | Tubers | **Constipation** | Decoction | Int. (a glass-once a day) | 3 | 0.04 | − | − |
|  |  |  | **Cough** | Boiled and powdered, then cooked with milk | Int. | 4 |  |  |  |
| *Dactylorhiza* *urvilleana* (Steud.) H. Baumann et Künkele, MARE 18362, 18409 | Sahlep, salep | Tubers | **Constipation** | Decoction | Int. (a glass-once a day) | 3 | 0.04 | (18,20)^b^ | − |
|  |  |  | **Cough** | Boiled, dried and powdered, then cooked with milk | Int. | 4 |  |  |  |
| *Orchis* *coriophora* L., MARE 18357 | Sahlep, salep | Tubers | **Constipation** | Decoction | Int. (a glass-once a day) | 3 | 0.04 | − | − |
|  |  |  | **Cough** | Boiled and powdered, then cooked with milk | Int. | 4 |  |  |  |
| ***Orchis* *morio*** L. subsp. ***morio***, MARE 18084, 18356, 18927 | Sahlep, salep | Tubers | **Constipation** | Decoction | Int. (a glass-once a day) | 3 | 0.04 | − | − |
|  |  |  | **Cough** | Boiled and powdered, then cooked with milk | Int. | 4 |  |  |  |
| *Orchis purpurea* Huds., MARE 18902 | Sahlep, salep | Tubers | **Constipation** | Decoction | Int. (a glass-once a day) | 3 | 0.04 | (8)^b^ | − |
|  |  |  | **Cough** | Boiled and powdered, then cooked with milk | Int. | 4 |  |  |  |
| ***Orchis simia*** Lam., MARE 18123, 18215, 18906, 18920 | Sahlep, salep | Tubers | **Constipation** | Decoction | Int. (a glass-once a day) | 3 | 0.04 | − | − |
|  |  |  | **Cough** | Boiled and powdered, then cooked with milk | Int. | 4 |  |  |  |
| ***Orchis tridentata*** Scop., MARE 18122, 18218 | Sahlep, salep | Tubers | **Constipation** | Decoction | Int. (a glass-once a day) | 3 | 0.04 | − | − |
|  |  |  | **Cough** | Boiled and powdered, then cooked with milk | Int. | 4 |  |  |  |
| **PAPAVERACEAE** | | | | | | | | | |
| *Chelidonium majus* L., MARE 18214, 18331, 18432, 18948, 19050 | Çizme otu, terme otu | Latex | Antifungal (in feet) | − | Ext. | 3 | 0.07 | Eczema(3,7,10,16,18,30,38,42,44)  Wound(10,16,31,34,38,43,44)  Antifungal (38,40,42)  (2,10,16,18,21,31,34,38,40,42,44)^b^ | Wound (34,52)  (5,19,24,33,34,47,52,53,54,55)^b^ |
|  |  |  | Wound | − | Ext. | 4 |  |  |  |
|  |  |  | Eczema | − | Ext. | 6 |  |  |  |
|  |  |  | **Diabetes** | − | Int. (1-2 drops swallowed) | 2 |  |  |  |
| *Papaver dubium* L., MARE 18141 | Gelincik | Flowers | **Shortness of breath** (in children) | Infusion | Int. | 3 | 0.02 | (20,37)^b^ | − |
| *Papaver rhoeas* L., MARE 18132, 18296, 18393, 18982 | Gelincik | Flowers | **Shortness of breath** (in children) | Infusion | Int. | 3 | 0.04 | (8,10,11,12,14,20,21, 38,41)^b^ |  |
|  |  | Aerial parts | **Rash** (in babies) | Waited in the water | Ext. (water is applied) | 4 |  |  |  |
| **PINACEAE** | | | | | | | | | |
| *Abies nordmanniana* subsp. *equi*-*trojani* (Asch. et Sint. ex Boiss.) Coode et Cullen, MARE 18224 [Syn.: *A. nordmanniana* subsp. *bornmuelleriana* (Mattf.) Coode et Cullen] | Göknar, köknar | Immature cones | Shortness of breath | Decoction | Int. | 9 | 0.28 | Shortness of breath (44)  (2,31,44,50,54)^b^ | Tuberculosis (52)  (52)^b^ |
|  |  |  | **Diabetes** | Decoction | Int. | 4 |  |  |  |
|  |  |  | **Prostate ailments** | Decoction | Int. | 4 |  |  |  |
|  |  |  | **Shortness of breath** | Molasses (boiled after adding sugar) | Int. (a spoon-twice a day) | 8 |  |  |  |
|  |  | Resin | Tuberculosis | Mixed with honey and butter | Int. (a spoon-twice a day) | 4 |  |  |  |
|  |  |  | **Immunostimulant** | Mixed with honey and butter | Int. (a spoon-twice a day) | 4 |  |  |  |
|  |  |  | **Shortness of breath** | − | Swallowed (2-3 pieces a day) | 5 |  |  |  |
|  |  |  | **Bronchitis** | − | Swallowed (2-3 pieces a day) | 5 |  |  |  |
|  |  |  | **Shortness of breath** | Mixed with honey | Int. (a spoon-twice a day) | 3 |  |  |  |
|  |  |  | **Bronchitis** | Mixed with honey | Int. (a spoon-twice a day) | 3 |  |  |  |
|  |  | Phloem (the inner layer of the stem bark, known as ‘soymuk’) | **Shortness of breath** | Eaten (in spring) | Int. | 6 |  |  |  |
| *Pinus nigra* subsp. *pallasiana* (Lamb.) Holmboe, MARE 18155, 18221, 18293, 18320, 18928, 19048 | Çam, kara çam | Fresh shoots | **Lung diseases** | Molasses (boiled after adding sugar) | Int. (a spoon-twice a day) | 3 | 0.78 | Wound (2,23,44)  Bronchitis (2,29,44)  Shortness of breath (44)  Tuberculosis (2)  (2,12,44)^b^ | − |
|  |  |  | **Cough** | Molasses (boiled after adding sugar) | Int. (a spoon-twice a day) | 4 |  |  |  |
|  |  |  | **Shortness of breath** | Molasses (boiled after adding sugar) | Int. (a spoon-twice a day) | 4 |  |  |  |
|  |  |  | **Expectorant** | Molasses (boiled after adding sugar) | Int. (a spoon-twice a day) | 4 |  |  |  |
|  |  |  | **Cough** | Decoction | Int. | 7 |  |  |  |
|  |  |  | **Shortness of breath** | Decoction | Int. | 4 |  |  |  |
|  |  | Immature cones | **Expectorant** | Molasses (boiled after adding sugar) | Int. (a spoon-twice a day) | 4 |  |  |  |
|  |  |  | **Cough** | Decoction | Int. | 5 |  |  |  |
|  |  |  | Bronchitis | Decoction | Int. | 4 |  |  |  |
|  |  |  | Tuberculosis | Decoction | Int. | 3 |  |  |  |
|  |  |  | **Shortness of breath** | Decoction | Int. | 27 |  |  |  |
|  |  |  | **Respiratory tract diseases** | Decoction | Int. | 3 |  |  |  |
|  |  |  | **Stomach ulcer** | Waited in honey for 20 days | Int. (a spoon-twice a day) | 2 |  |  |  |
|  |  | Resin | **Halitosis** | Chewed | Ext. | 3 |  |  |  |
|  |  |  | **Dental problems** | Chewed | Ext. | 3 |  |  |  |
|  |  |  | **Respiratory tract diseases** | Mixed with honey | Int. (a spoon-twice a day) | 3 |  |  |  |
|  |  |  | Tuberculosis | Mixed with honey | Int. (a spoon-twice a day) | 2 |  |  |  |
|  |  |  | **Cracked hand-heels** | − | Ext. | 3 |  |  |  |
|  |  |  | Wound | Heated | Ext. | 12 |  |  |  |
|  |  |  | Wound | Heated (mixed with wax and butter) | Ext. | 4 |  |  |  |
|  |  | Phloem (the inner layer of the stem bark, known as ‘soymuk’) | Bronchitis | Eaten (in spring) | Int. | 3 |  |  |  |
|  |  |  | Tuberculosis | Eaten (in spring) | Int. | 2 |  |  |  |
|  |  |  | Shortness of breath | Eaten (in spring) | Int. | 19 |  |  |  |
|  |  |  | Cough | Eaten (in spring) | Int. | 3 |  |  |  |
|  |  |  | **Abdominal pain** | Eaten (in spring) | Int. | 3 |  |  |  |
|  |  |  | **Cough** | Decoction | Int. | 4 |  |  |  |
|  |  |  | **Shortness of breath** | Decoction | Int. | 4 |  |  |  |
|  |  |  | Bronchitis | Decoction | Int. | 4 |  |  |  |
|  |  | Wood (decayed) | **Rash** (in the armpit in babies) | − | Ext. | 1 |  |  |  |
|  |  | Wood ash | **Antifungal** (in feet) | − | Ext. | 2 |  |  |  |
| *Pinus sylvestris* var. *hamata* Steven, MARE 18174, 18226, 18364, 18407, 18938 | Çam, sarı çam | Fresh shoots | Lung diseases | Molasses (boiled after adding sugar) | Int. (a spoon-twice a day) | 3 | 0.75 | Cough (4,34,40)  Bronchitis (3,4,17,20,34,40,45)  Shortness of breath (10,14,38,39,45)  Expectorant (20,40)  Lung diseases (34,40)  Tuberculosis (2,4)  Stomach ulcer (4)  Wound (2,10,23,34,38,40,45)  Inflamed wound (20)  (2,3,4,10,17,20,40,42,45,50)^b^ | Cough (19,34)  Bronchitis (34)  Lung diseases (34)  Wound (34)  (19,34)^b^ |
|  |  |  | Cough | Molasses (boiled after adding sugar) | Int. (a spoon-twice a day) | 4 |  |  |  |
|  |  |  | Shortness of breath | Molasses (boiled after adding sugar) | Int. (a spoon-twice a day) | 4 |  |  |  |
|  |  |  | Expectorant | Molasses (boiled after adding sugar) | Int. (a spoon-twice a day) | 4 |  |  |  |
|  |  |  | **Cough** | Decoction | Int. | 7 |  |  |  |
|  |  |  | **Shortness of breath** | Decoction | Int. | 4 |  |  |  |
|  |  | Immature cones | Expectorant | Molasses (boiled after adding sugar) | Int. (a spoon-twice a day) | 4 |  |  |  |
|  |  |  | Bronchitis | Decoction | Int. | 4 |  |  |  |
|  |  |  | Shortness of breath | Decoction | Int. | 19 |  |  |  |
|  |  |  | Shortness of breath | Molasses (boiled after adding sugar) | Int. (a spoon-twice a day) | 3 |  |  |  |
|  |  |  | Tuberculosis | Decoction | Int. | 3 |  |  |  |
|  |  |  | Stomach ulcer | Waited in honey for 20 days | Int. (a spoon-twice a day) | 2 |  |  |  |
|  |  | Resin | **Cracked hand-heels** | − | Ext. | 3 |  |  |  |
|  |  |  | **Halitosis** | Chewed | Ext. | 3 |  |  |  |
|  |  |  | **Dental problems** | Chewed | Ext. | 3 |  |  |  |
|  |  |  | Wound | Heated | Ext. | 6 |  |  |  |
|  |  |  | Inflamed wound | − | Ext. | 3 |  |  |  |
|  |  |  | **Wound** | Heated (mixed with wax and butter) | Ext. | 4 |  |  |  |
|  |  | Phloem (the inner layer of the stem bark, known as ‘soymuk’) | **Bronchitis** | Eaten (in spring) | Int. | 3 |  |  |  |
|  |  |  | Tuberculosis | Eaten (in spring) | Int. | 2 |  |  |  |
|  |  |  | Shortness of breath | Eaten (in spring) | Int. | 4 |  |  |  |
|  |  |  | Cough | Eaten (in spring) | Int. | 10 |  |  |  |
|  |  |  | **Cough** | Decoction | Int. | 6 |  |  |  |
|  |  |  | **Shortness of breath** | Decoction | Int. | 25 |  |  |  |
|  |  |  | **Bronchitis** | Decoction | Int. | 4 |  |  |  |
|  |  | Wood (decayed) | **Rash** (in the armpit in babies) | − | Ext. | 1 |  |  |  |
|  |  | Wood ash | **Antifungal** (in feet) | − | Ext. | 2 |  |  |  |
| **PLANTAGINACEAE** | | | | | | | | | |
| *Plantago* *lanceolata* L., MARE 18121, 18147, 18191, 18235, 18349, 18441, 18915, 18996 | Damar otu, siğil otu, siğil yaprağı, sinir otu, sinir yaprağı | Leaves | Wound | − | Ext. | 92 | 0.58 | Burn (3)  Wound (3,10,18,21,34,38,40,44)  Acne (40)  Diabetes (40,44)  Cough (10,34,44) (3,6,8,10,12,14,21,34,38,40,43,44)^b^ | Wound (34,54)  Cough (34)  (34,54)^b^ |
|  |  |  | Wound | Crushed | Ext. (wrapped in a cloth) | 5 |  |  |  |
|  |  |  | Acne | − | Ext. | 2 |  |  |  |
|  |  |  | Burn | − | Ext. | 2 |  |  |  |
|  |  |  | **Antifungal** | − | Ext. (put into shoes) | 1 |  |  |  |
|  |  |  | **Sore throat** | − | Int. | 2 |  |  |  |
|  |  |  | **Stomach ulcer** | − | Int. | 2 |  |  |  |
|  |  |  | **Prostate ailments** | Decoction | Int. | 1 |  |  |  |
|  |  |  | Diabetes | Decoction | Int. | 2 |  |  |  |
|  |  |  | Cough | Decoction | Int. | 5 |  |  |  |
| *Plantago* *major* subsp. *intermedia* (Gilib.) Lange, MARE 18442 | Damar otu, siğil otu, siğil yaprağı, sinir otu, sinir yaprağı | Leaves | **Wound** | − | Ext. | 92 | 0.58 | Wound (10)  (2,7,10,27)^b^ | − |
|  |  |  | Wound | Crushed | Ext. (wrapped in a cloth) | 5 |  |  |  |
|  |  |  | **Acne** | − | Ext. | 2 |  |  |  |
|  |  |  | **Burn** | − | Ext. | 2 |  |  |  |
|  |  |  | **Antifungal** | − | Ext. (put into shoes) | 1 |  |  |  |
|  |  |  | **Sore throat** | − | Int. | 2 |  |  |  |
|  |  |  | **Stomach ulcer** | − | Int. | 2 |  |  |  |
|  |  |  | **Prostate ailments** | Decoction | Int. | 1 |  |  |  |
|  |  |  | **Diabetes** | Decoction | Int. | 2 |  |  |  |
|  |  |  | **Cough** | Decoction | Int. | 5 |  |  |  |
| *Plantago* *major* L., MARE 18385, 18400, 18997 [Syn.: *P.* *major* L. subsp. *major*] | Damar otu, siğil otu, siğil yaprağı, sinir otu, sinir yaprağı | Leaves | Wound | − | Ext. | 92 | 0.58 | Diabetes (3,10,14,38,44)  Wound (3,4,10,16,17,18,21, 34,38,39,40,44,51)  Cough (16,34,38,40,44,45)  Sore throat (4,34,38,51)  Burn (38)  Stomach ulcer (34)  Prostate ailments (9, 38)  (3,4,6,8,9,11,14,16,17,20,21,25,27,29,30,32,37,38,39,40,42,43,44,45,46,47,49,50)^b^ | Wound (19,34,52,53,54,55)  Acne (19)  Cough (19,34,52,53)  Sore throat (34)  Stomach ulcer (34)  (19,33,34,47,52,53,54,55)^b^ |
|  |  |  | Wound | Crushed | Ext. (wrapped in a cloth) | 5 |  |  |  |
|  |  |  | Acne | − | Ext. | 2 |  |  |  |
|  |  |  | Burn | − | Ext. | 2 |  |  |  |
|  |  |  | **Antifungal** | − | Ext. (put into shoes) | 1 |  |  |  |
|  |  |  | Sore throat | − | Int. | 2 |  |  |  |
|  |  |  | Stomach ulcer | − | Int. | 2 |  |  |  |
|  |  |  | Prostate ailments | Decoction | Int. | 1 |  |  |  |
|  |  |  | Diabetes | Decoction | Int. | 2 |  |  |  |
|  |  |  | Cough | Decoction | Int. | 5 |  |  |  |
| **POACEAE** | | | | | | | | | |
| *Cynodon dactylon* (L.) Pers., MARE 18236 [Syn.: *C.* *dactylon* var. *villosus* Regel] | Ayrık | Rhizomes | Rheumatic pain | Decoction | Int. (a glass-twice a day) | 2 | 0.03 | Diuretic (1,10,11)  Rheumatic pain (26,28)  (1,4,10,11,12,16,26,27,28,29,31,43,44)^b^ | − |
|  |  |  | Diuretic | Decoction | Int. (a glass-twice a day) | 4 |  |  |  |
| ***Hordeum distichon*** L.^a^, MARE 18444 | Arpa | Fruits | **Bone fracture** | Crushed and boiled | Ext. (as poultice) | 1 | 0.02 | − | − |
|  |  |  | **Dislocation** | Crushed and boiled | Ext. (as poultice) | 1 |  |  |  |
|  |  |  | **Urinary tract infection** | Decoction | Int. | 1 |  |  |  |
| *Triticum aestivum* L.^a^, MARE 18455 | Buğday | Fruits | **Diarrhoea** | Crushed | Int. (a handful) | 2 | 0.01 | (10)^b^ | − |
| **POLYGONACEAE** | | | | | | | | | |
| *Polygonum cognatum* Meisn., MARE 18451, 19000 | Madımak | Aerial parts | Constipation | Cooked | Int.(eaten) | 4 | 0.02 | Constipation (20)  (20,34,44,47,51)^b^ | (34)^b^ |
| *Rumex crispus* L., MARE 18151, 18271, 18396, 18445, 18955, 18979, 19043 | Acımık otu, kuzukulağı, sığırkuyruğu | Leaves | **Diabetes** | − | Int. (eaten) | 4 | 0.04 | Constipation (20)  (4,8,10,14,15,20,34,37,44,47)^b^ | Constipation (54)  (34,53)^b^ |
|  |  |  | Constipation | − | Int. (eaten) | 3 |  |  |  |
| **PRIMULACEAE** | | | | | | | | | |
| *Primula* *vulgaris* Huds., MARE 18457, 18843, 18866 [Syn.: *P. vulgaris* Huds. subsp. *vulgaris*] | Menevşe | Leaves | **Analgesic** | Heated | Ext. (wrapped in a cloth) | 2 | 0.01 | (1,13,18,21,40)^b^ | − |
| **ROSACEAE** | | | | | | | | | |
| *Crataegus azarolus* var. *pontica* (K.Koch) K.I.Chr., MARE 18403 [Syn.: *C.* *pontica* K. Koch] | Alıç, öküzgötü, yemişen | Fruits | **Cardiovascular system diseases** | Decoction | Int. | 9 | 0.10 | (34)^b^ | (34)^b^ |
|  |  | Flowering branches | **Cardiovascular system diseases** | Infusion | Int. | 5 |  |  |  |
|  |  |  | **Common cold** | Decoction | Int. | 2 |  |  |  |
|  |  | Leaves | **Common cold** | Decoction | Int. | 5 |  |  |  |
| *Crataegus monogyna* Jacq., MARE 18287, 18310, 18951 [Syn.: *C. monogyna* Jacq. subsp. *monogyna*] | Alıç, öküzgötü, yemişen | Fruits | Cardiovascular system diseases | Decoction | Int. | 9 | 0.10 | Cardiovasc. sys. dis. (8,10,26,44)  (8,10,18,26,34,41,44,47)^b^ | Cardiovasc. sys. dis. (5,19,34)  (5,19,34)^b^ |
|  |  | Flowering branches | Cardiovascular system diseases | Infusion | Int. | 5 |  |  |  |
|  |  |  | **Common cold** | Decoction | Int. | 2 |  |  |  |
|  |  | Leaves | **Common cold** | Decoction | Int. | 5 |  |  |  |
| *Crataegus orientalis* Pall. ex M.Bieb. subsp. *orientalis*, MARE 18294 [Syn.: *C.* *orientalis* var. *obtusata* Browicz] | Alıç, öküzgötü, yemişen | Fruits | Cardiovascular system diseases | Decoction | Int. | 9 | 0.10 | Cardiovasc. sys. dis. (9,20,44)  (20,25,37,34,38,44)^b^ | Cardiovasc. sys. dis. (34)  (34)^b^ |
|  |  | Flowering branches | Cardiovascular system diseases | Infusion | Int. | 5 |  |  |  |
|  |  |  | **Common cold** | Decoction | Int. | 2 |  |  |  |
|  |  | Leaves | **Common cold** | Decoction | Int. | 5 |  |  |  |
| *Crataegus rhipidophylla* Gand. var. *rhipidophylla*, MARE 18113, 18973 [Syn.: *C. curvisepala* Lindm.] | Alıç, öküzgötü, yemişen | Fruits | Cardiovascular system diseases | Decoction | Int. | 9 | 0.10 | Cardiovascular system diseases (40)  Common cold (40)  (40)^b^ | Cardiovasc. sys. dis. (22,52)  (55)^b^ |
|  |  | Flowering branches | Cardiovascular system diseases | Infusion | Int. | 5 |  |  |  |
|  |  |  | Common cold | Decoction | Int. | 2 |  |  |  |
|  |  | Leaves | Common cold | Decoction | Int. | 5 |  |  |  |
| *Crataegus* *tanacetifolia* (Poir.) Pers., MARE 18116, 18144, 18358 | Alıç, öküzgötü, yemişen | Fruits | Cardiovascular system diseases | Decoction | Int. | 9 | 0.10 | Cardiovasc. sys. dis. (20)  (20,29)^b^ | − |
|  |  | Flowering branches | Cardiovascular system diseases | Infusion | Int. | 5 |  |  |  |
|  |  |  | **Common cold** | Decoction | Int. | 2 |  |  |  |
|  |  | Leaves | **Common cold** | Decoction | Int. | 5 |  |  |  |
| *Cydonia* *oblonga* Mill.^a^, MARE18163, 18333, 18950 | Ayva | Leaves | Cough | Decoction | Int. | 40 | 0.28 | Cough (4,6,8,9,10,11,14,15,26,27,29,31,38,39,40,43)  Common cold (4,10,11,12,20,26,27,29,31,38,39,40,43,44)  Shortness of breath (29,31)  (3,4,6,7,8,9,10,12,13,14,15,17,20,27,29,31,32, 38,39,40,41,43,44)^b^ | (22,24,33,52)^b^ |
|  |  |  | Common cold | Decoction | Int. | 10 |  |  |  |
|  |  |  | Shortness of breath | Infusion | Int. | 5 |  |  |  |
| *Malus sylvestris* (L.) Mill., MARE 18853, 19023 [Syn.: *M. sylvestris* subsp. *orientalis* (A. Uglitzkich) Browicz var. *orientalis*] | Acuk | Fruits | Diabetes | − | Int.(eaten 3 a day) | 4 | 0.02 | Diabetes (10,37,44)  (10,17,37)^b^ | (15)^b^ |
| *Prunus avium* (L.) L., MARE 18370, 18942, 18990 [Syn.: *Cerasus avium* (L.) Moench] | Kuş kirazı, kiraz | Fruit stalks | Diuretic | Infusion | Int. (a glass-once a day) | 4 | 0.02 | Diuretic (2,7,10,26,29,31,40)  (3,8,10,11,12,13,15,17,20,26,27,29,31,34,36,38,40,43,44)^b^ | (34)^b^ |
| *Prunus divaricata* subsp. *divaricata* Ledeb., MARE 18887, 18991 | Ala erik, erik, örük | Fruits | Constipation | − | Int. (eaten a handful a day) | 2 | 0.01 | Constipation (44)  (34,38,44)^b^ | (34,24,47,54,55)^b^ |
| *Pyrus elaeagnifolia* subsp. *elaeagnifolia* Pall., MARE 18112, 18220, 18392 | Ahlat, kel ahlat | Fruits | **Cough** | Molasses | Int. (a spoon-twice a day) | 19 | 0.20 | Diarrhoea (2,25,29,44)  (10,23,25,44)^b^ | − |
|  |  |  | **Common cold** | Molasses | Int. (a spoon-twice a day) | 10 |  |  |  |
|  |  |  | **Shortness of breath** | Molasses | Int. (a spoon-twice a day) | 5 |  |  |  |
|  |  |  | **Icterus** | Molasses | Int. (a spoon-twice a day) | 2 |  |  |  |
|  |  |  | Diarrhoea | Crushed | Int. (eaten 3-4 a day) | 3 |  |  |  |
| *Rosa canina* L., MARE 18148, 18183, 18288, 18342, 18387, 18907, 19047 | Kışburnu, kuşburnu | Fruits | Cough | Decoction | Int. | 16 | 0.32 | Common cold (9,11,14,15,16,18,21,25,29,32,38,39,40,44,51)  Cough (8,10,14,21,29,34,40,44)  Eczema (6,8,10,32)  Haemorrhoids (2,4,6,8,9,11,29,34,38,44,50)  Sedative (25)  (2,4,6,8,9,10,11,14,15,16,18,25,27,29,32,34,38,39,40,41,44,51)^b^ | Haemorrhoids (34)  Common cold (34)  Cough (34)  Urinary sys. dis. (55)  (22,34,53,54,55)^b^ |
|  |  |  | Urinary system diseases | Decoction | Int. | 2 |  |  |  |
|  |  |  | Common cold | Infusion | Int. | 36 |  |  |  |
|  |  |  | Sedative | Infusion | Int. | 3 |  |  |  |
|  |  | Roots | Eczema | Decoction | Int. | 2 |  |  |  |
|  |  |  | Haemorrhoids | Decoction | Int. | 4 |  |  |  |
| *Rubus canescens* DC. var. *canescens*, MARE 18343, 18411 [*R. canescens* DC.] | Böğürtlen, kır böğürtleni | Leaves | Wound | Powdered | Ext. | 3 | 0.10 | Wound (16,39)  Haemorrhoids (4,20)  Diabetes(41) (4,10,14,16,20,21,23,32,39,40,41)^b^ | − |
|  |  |  | **Wound** | Powdered and mixed with olive oil | Ext. | 1 |  |  |  |
|  |  | Roots | **Diabetes** | Decoction (cut into little pieces) | Int. | 9 |  |  |  |
|  |  |  | Haemorrhoids | Decoction (cut into little pieces) | Int. | 4 |  |  |  |
|  |  | Young shoots | Diabetes | Decoction | Int. | 2 |  |  |  |
| *Rubus canescens* var. *glabratus* (Godr.) P.H. Davis et Meikle, MARE 18345 [*R. canescens* DC.] | Böğürtlen, kır böğürtleni | Leaves | Wound | Powdered | Ext. | 3 | 0.10 | Wound (10)  Diabetes (8)  Haemorrhoids (11) (6,8,10,11)^b^ | − |
|  |  |  | **Wound** | Powdered and mixed with olive oil | Ext. | 1 |  |  |  |
|  |  | Roots | Diabetes | Decoction (cut into little pieces) | Int. | 9 |  |  |  |
|  |  |  | Haemorrhoids | Decoction (cut into little pieces) | Int. | 4 |  |  |  |
|  |  | Young shoots | Diabetes | Decoction | Int. | 2 |  |  |  |
| *Rubus hirtus* Waldst. et Kit., MARE 18373, 19071 | Böğürtlen, kır böğürtleni | Leaves | Wound | Powdered | Ext. | 3 | 0.10 | Wound (13,40)  Diabetes (2)  Haemorrhoids (3,40)  (2,10,13,40)^b^ | − |
|  |  |  | **Wound** | Powdered and mixed with olive oil | Ext. | 1 |  |  |  |
|  |  | Roots | Diabetes | Decoction (cut into little pieces) | Int. | 9 |  |  |  |
|  |  |  | Haemorrhoids | Decoction (cut into little pieces) | Int. | 4 |  |  |  |
|  |  | Young shoots | **Diabetes** | Decoction | Int. | 2 |  |  |  |
| *Rubus sanctus* Schreb., MARE 18268, 18317, 18414, 19068 | Böğürtlen, dağ böğürtleni, kır böğürtleni | Leaves | Wound | Powdered | Ext. | 3 | 0.10 | Wound (4,6,12,16,29)  Diabetes (2,9,15)  Haemorrhoids (4,9)  (4,6,8,9,11,12,14,15,16,29,44)^b^ | − |
|  |  |  | **Wound** | Powdered and mixed with olive oil | Ext. | 1 |  |  |  |
|  |  | Roots | Diabetes | Decoction (cut into little pieces) | Int. | 9 |  |  |  |
|  |  |  | Haemorrhoids | Decoction (cut into little pieces) | Int. | 4 |  |  |  |
|  |  | Young shoots | **Diabetes** | Decoction | Int. | 2 |  |  |  |
| *Sorbus domestica* L.^a^, MARE 18945 | Üvez | Fruits | Common cold | − | Int. | 3 | 0.07 | Common cold (12)  Diabetes (10,44)  (4,6,9,10,44,50)^b^ | − |
|  |  | Leaves | Common cold | Decoction | Int. | 3 |  |  |  |
|  |  |  | **Shortness of breath** | Decoction | Int. | 3 |  |  |  |
|  |  |  | Diabetes | Infusion | Int. | 4 |  |  |  |
| **SALICACEAE** | | | | | | | | | |
| ***Populus* *nigra*** L., MARE 18186, 18850, 18888 [Syn.: *P. nigra* L. subsp. *nigra*] | Kavak | Leaves | **Rheumatism** | Infusion | Int. | 2 | 0.01 | − | − |
| *Salix alba* L., MARE 18300, 18881, 19034 | Söğüt | Branch bark | Headache | Chewed | Int. | 1 | 0.04 | Headache (10,15,20)  (4,9,10,20,21,32,44)^b^ | (22,54)^b^ |
|  |  | Leaves | **Sunstroke** (in children) | − | Ext. (wrapped in a cloth around the head and nape) | 2 |  |  |  |
|  |  |  | **Fever** (in children) | − | Ext. (wrapped in a cloth around the body) | 5 |  |  |  |
| *Salix* x *fragilis* L., MARE 18389 [Syn.: *S.* *fragilis* L.] | Söğüt | Leaves | **Fever** (in children) | − | Ext. (wrapped in a cloth around the body) | 5 | 0.03 | (37)^b^ | − |
| **SANTALACEAE** | | | | | | | | | |
| *Viscum album* L., MARE 18115, 18159, 18849, 18929 [Syn.: *V. album* L. subsp. *album*] | Hurç, purç, ökse otu | Leafy branches | Diabetes | Decoction | Int. (a glass-twice a day) | 8 | 0.07 | Urinary sys. dis. (34)  Diabetes (9,16,20,34,44)  (2,9,10,18,21,28,34,42,43,44,45)^b^ | Urinary sys. dis. (34)  Diabetes (34)  (22,34,47,53,54,55)^b^ |
|  |  |  | Urinary system diseases | Decoction | Int. | 3 |  |  |  |
|  |  |  | **Skin diseases** | Infusion | Ext. | 2 |  |  |  |
| *Viscum album* subsp. *austriacum* (Wiesb.) Vollm., MARE 18454 | Çam purcu, hurç, purç, ökse otu | Leafy branches | Diabetes | Decoction | Int. | 8 | 0.08 | Diabetes (29,40)  (29,32,40)^b^ | − |
|  |  |  | **Urinary system diseases** | Decoction | Int. | 3 |  |  |  |
|  |  |  | **Shortness of breath** | Decoction | Int. | 2 |  |  |  |
|  |  |  | **Skin diseases** | Infusion | Ext. | 2 |  |  |  |
| **SCROPHULARIACEAE** | | | | | | | | | |
| *Verbascum speciosum* Schrad., MARE 18284, 19013 | − | Leaves | **Sprain** | Boiled | Ext. (wrapped in a cloth) | 2 | 0.01 | (16)^b^ | − |
| **SOLANACEAE** | | | | | | | | | |
| *Hyoscyamus niger* L., MARE 18140, 18277, 18437, 18439, 18884, 18931, 18989 | Caba otu, çömlek otu | Seeds | Eye diseases | Thrown into boiling water | Ext. (eye is exposed to the vapour) | 7 | 0.12 | Eye diseases (2,3,4,44,49)  (2,3,4,10,14,20,31,34,37,40,41,44,49,51)^b^ | (34,53,54)^b^ |
|  |  |  |  | Burnt on the ember | Ext. (eye is exposed to the smoke) | 17 |  |  |  |
| **URTICACEAE** | | | | | | | | | |
| *Urtica dioica* L., MARE 18161, 18269, 18386, 19069 | Isırgan | Aerial parts | Gynecological diseases | Decoction | Int. (a glass-once a day) | 1 | 0.22 | Rheumatism (1,2,3,6,9,25,26,34,38,39,40,43,44,46,51)  Diabetes (2,6,9,11,25,34,39,40,44)  Hair loss(34)  Haemorrhoids (6,11,25,26,38)  Prostate ailments (11, 38)  Gynecological diseases (38)  (1,3,6,9,11,12,13,25,26,32,34,38,39,40,43,44,47,50)^b^ | Rheumatism (19,34)  Diabetes (19)  Hair loss(34)  Haemorrhoids(34)  (19,24,34,47,52,53,54,55)^b^ |
|  |  |  | Diabetes | Decoction | Int. (a glass-twice a day) | 3 |  |  |  |
|  |  |  | **Strengthening** | Decoction | Int. (a glass-once a day) | 1 |  |  |  |
|  |  |  | Haemorrhoids | Decoction | Int. (a glass-once a day) | 2 |  |  |  |
|  |  |  | Prostate ailments | Decoction | Int. (a glass-once a day) | 3 |  |  |  |
|  |  |  | **Urinary system diseases** | Decoction | Int. (a glass-once a day) | 3 |  |  |  |
|  |  |  | Rheumatism | Decoction | Ext. (knees are immersed in decoction) | 4 |  |  |  |
|  |  |  | Hair loss | Decoction | Ext. (as rinse water) | 2 |  |  |  |
|  |  |  | Diabetes | Cooked | Int. | 2 |  |  |  |
|  |  |  | **Strengthening** | Cooked | Int. | 2 |  |  |  |
|  |  |  | **Gynecological diseases** | Cooked | Int. | 1 |  |  |  |
|  |  |  | Rheumatism | − | Ext. (hit to the knees) | 11 |  |  |  |
| *Urtica urens* L., MARE 18282 | Isırgan | Aerial parts | **Gynecological diseases** | Decoction | Int. (a glass-once a day) | 1 | 0.22 | Haemorrhoids (15)  Rheumatism (2,15,50)  (14,15)^b^ | − |
|  |  |  | **Diabetes** | Decoction | Int. (a glass-twice a day) | 2 |  |  |  |
|  |  |  | **Strengthening** | Decoction | Int. (a glass-once a day) | 3 |  |  |  |
|  |  |  | Haemorrhoids | Decoction | Int. (a glass-once a day) | 3 |  |  |  |
|  |  |  | **Prostate ailments** | Decoction | Int. (a glass-once a day) | 4 |  |  |  |
|  |  |  | **Urinary system diseases** | Decoction | Int. (a glass-once a day) | 2 |  |  |  |
|  |  |  | Rheumatism | Decoction | Ext. (knees are immersed in decoction) | 2 |  |  |  |
|  |  |  | **Hair loss** | Decoction | Ext. (as rinse water) | 2 |  |  |  |
|  |  |  | **Diabetes** | Cooked | Int. | 1 |  |  |  |
|  |  |  | **Strengthening** | Cooked | Int. | 11 |  |  |  |
|  |  |  | **Gynecological diseases** | Cooked | Int. | 1 |  |  |  |
|  |  |  | Rheumatism | − | Ext. (hit to the knees) | 2 |  |  |  |

Rpt.; Reports. Int.; Internal use. Ext.; External use. ^a^ Cultivated plant. ^b^ Different usage. Obs.: Observation. New plant records and new uses were marked as bold.

(1) Sezik et al., 1992; (2) Fujita et al., 1995; (3) Yazicioglu and Tuzlaci, 1995; (4) Yesilada et al., 1999; (5) Ivancheva and Stantcheva, 2000; (6) Tuzlaci and Tolon, 2000; (7) Uzun et al., 2004; (8) Ecevit Genc and Ozhatay 2006; (9) Ezer and Mumcu Arisan, 2006; (10) Kultur, 2007; (11) Tuzlaci and Alparslan, 2007; (12) Koyuncu et al., 2009; (13) Dogru Koca and Yildirimli, 2010; (14) Tuzlaci et al., 2010; (15) Bulut, 2011; (16) Kizilarslan and Ozhatay, 2012; (17) Sagiroglu et al., 2012; (18) Sarac et al., 2013; (19) Koleva et al., 2015; (20) Korkmaz and Karakurt, 2015; (21) Polat et al., 2015; (22) Bussmann et al., 2016a; (23) Akbulut et al., 2017; (24) Bussmann et al., 2017a; (25) Eminagaoglu et al., 2017; (26) Gunes, 2017; (27) Karci et al., 2017; (28) Kartal and Gunes, 2017; (29) Yesilyurt et al., 2017; (30) Aydin and Yesil, 2018; (31) Gurbuz et al., 2019; (32) Karakose et al., 2019; (33) Bussmann et al., 2020a; (34) Kazanci et al., 2020; (35) Guler et al., 2021; (36) Gurdal and Ozturk, 2021; (37) Kadıoglu et al., 2021; (38) Sener et al., 2022; (39) Akbulut et al., 2022; (40) Karakose, 2022a; (41) Turkan et al., 2006; (42) Cansaran et al., 2007; (43) Akbulut and Ozkan, 2014; (44) Tuttu, 2017; (45) Badem et al., 2018; (46) Ergul Bozkurt, 2021; (47) Kazanci et al., 2021; (48) Akbulut, 2022; (49) Sezik et al., 1991; (50) Sezik et al., 1997; (51) Akgul, 2008; (52) Bussmann et al., 2016b; (53) Bussmann et al., 2017b; (54) Jakeli et al., 2018; (55) Bussmann et al., 2020b.
