# Supplementary material for: An Ethnobotanical study of medicinal plants in Taşköprü (Kastamonu–Turkey)
Source: Front Pharmacol. 2022 Oct 20;13:984065. doi: 10.3389/fphar.2022.984065 (PMC9630845; doi:10.3389/fphar.2022.984065)
Supplement: Supplementary file 2 [file Table2.docx]

**Supplementary Table S2. Questionnaire Form**

1. Participant's name and surname

2. Participant's age and sex

3. Participant's telephone and address

4. Participant's educational level

5. Interview date

6. Participant's place of residence

7. Participant's duration of residence

8. Plant’s local name

9. Human / animal health

10. Ailments treated /therapeutic effect

11. Plant part used

12. Preparation

13. Administration

14. Dosage

15. Length of treatment

16 Age group of patients (baby, child, adult)

17. Side effect

18. Other ethnobotanical uses
